# One-year efficacy and safety of single or one to three weekly injections of hylan G-F 20 for knee osteoarthritis: a systematic literature review and meta-analysis

**DOI:** 10.1007/s10067-020-05477-7

**Published:** 2020-10-27

**Authors:** Orazio De Lucia, Joerg Jerosch, Sophie Yoon, Tobias Sayre, Wilson Ngai, Georgios Filippou

**Affiliations:** 1 Clinical Rheumatology Unit, Department of Rheumatology and Medical Sciences, ASST Centro Traumatologico Ortopedico G. Pini-CTO, Milan, Italy; 2 Orthopedic Department, Johanna Étienne Hospital, Am Hasenberg 46, 41462 Neuss, Germany; 3 Doctor Evidence, Santa Monica, CA USA; 4 Global Medical Affairs, Sanofi, Bridgewater, NJ USA; 5 ASST Fatebenefratelli, University Hospital “Luigi Sacco”, Rheumatology Unit, Via Giovanni Battista Grassi, 74, 20157 Milan, Italy

**Keywords:** Hylan G-F 20, Injections, Knee, Osteoarthritis, Osteoarthritis therapy

## Abstract

The aim of this study was to evaluate the long-term efficacy and safety of single or 1–3 weekly injections of hylan G-F 20 at 1 year following the first injection for knee osteoarthritis (OA). Searches were conducted in PubMed/MEDLINE, Embase, and CENTRAL and included relevant conference proceedings (January 1, 1995–August 17, 2020). Randomized controlled trials (RCTs), non-randomized trials, and observational studies investigating 1-year efficacy and safety of 1–3 weekly injections or single hylan G-F 20 injection for knee OA were included. Primary outcomes were WOMAC pain, physical function, and stiffness. Meta-analyses of RCTs and non-randomized studies were conducted separately. Our search identified 24 eligible studies. Hylan G-F 20, in the meta-analyses of RCTs, showed statistically significant improvement in WOMAC pain (SMCC − 0.98, 95% CI − 1.50, − 0.46), physical function (SMCC − 1.05, 95% CI − 1.28, − 0.83), and stiffness (SMCC − 1.07, 95% CI −1.28, −0.86). Improvement was also seen for VAS pain, SF-36 MCS (mental component summary), and SF-36 PCS (physical component summary). Analyses of non-randomized studies showed similar efficacy estimates. There were no significant differences in efficacy based on injection schedule, nor between RCT and non-randomized studies. Rates of adverse events (AEs) were low for most types of AEs. Hylan G-F 20 (either as single or 1–3 weekly injections) showed improvement in 1-year efficacy outcomes in comparison to baseline and was generally well tolerated. While further research will inform the medical field regarding viscosupplementation treatment options for knee OA, these findings show that hylan G-F 20 at both frequencies/dosages are efficacious and generally well tolerated for long-term use.

**Supplementary Information:**

The online version contains supplementary material available at 10.1007/s10067-020-05477-7.

## Introduction

Hyaluronic acid (HA), a polymer of B-D glucuronic acid and beta-D N-acetylglucosamine, is a naturally occurring substance in the synovial fluid and cartilage [1]. It confers the rheologic and viscoelastic properties of the synovial fluid and enables the synovial fluid to lubricate the joints as well as act as a shock absorber [2]. Introducing exogenous HA in the joint space may restore some of the viscoelastic and protective properties of the fluid space and ameliorate the cartilage degradation seen in osteoarthritis (OA) [3]. Early meta-analyses supported the efficacy and safety of HA for the treatment of knee OA [4].

Hylan G-F 20 is an HA preparation consisting of hylan A, a 6000 kDa HA, and hylan B, a cross-linked derivative of natural HA [5, 6]. There are two hylan G-F 20 formulations: a single-shot (wherein a higher volume is administered) and the once weekly x 3 approach (wherein a lower volume is administered across multiple injections). An early Cochrane review found that hylan G-F 20 significantly improved pain and movement relative to placebo, significantly improved pain but not function relative to NSAIDS, and significantly improved pain as well as function when added to standard of care [7]. Despite mixed results from head-to-head trials comparing different HA formulations [8–11], many of the more recent meta-analyses have taken a broader focus by combining multiple HA formulations and subsequently found lower efficacy estimates [12] and higher rates of adverse events [13]. A return to more focused meta-analyses will likely benefit this field given the lack of consensus among current meta-analyses [14] and even practice guidelines involving the use of HA for the treatment of knee OA [15, 16].

Reviews comparing hylan G-F 20 with intra-articular corticosteroids (triamcinolone hexacetonide and betamethasone) show that although intra-articular corticosteroids provide more relief than hylan G-F 20 within the first few weeks after treatment, hylan G-F 20 provides comparable or greater sustained pain relief over time [7, 17]. Additionally, several observational studies suggest that hylan G-F 20 could delay the need for arthroplasty [18, 19]. Previous meta-analytic investigations show the efficacy and safety of HA in the treatment of knee OA at 6 months [20], and some studies show that the impact of treatment with hylan G-F 20 can be present up to a year after treatment [21, 22]. Even so, the efficacy of HA for treatment of OA of the knee is a point of disagreement even among published meta-analyses and guidelines [23]. One recent review concluded that “there is considerable between-product, between-variable and time-dependent variability in the clinical response” to HA injections [24]. The current study contributes to the field by conducting a more focused review on a single well-studied HA formulation with a protocol-defined follow-up assessment time. The aim of this systematic review and meta-analysis was to evaluate the efficacy and safety of hylan G-F 20 at 1 year following first injection.

## Methods

### Literature search and screening

This systematic literature review was not registered with the international prospective register of systematic reviews (PROSPERO). The screening and study selection processes were conducted and presented according to the Preferred Reporting Items for Systematic Reviews and Meta-Analyses (PRISMA) guidelines [25]. Standard methodology for conducting systematic reviews provided by the Cochrane Handbook for Systematic Reviews of Interventions was followed [26]. A standardized review protocol using the PICO framework (*p*opulation, *i*nterventions, *c*omparators, *o*utcomes) was used to define the criteria for the literature search and screening (Supplementary Material Table S0). Eligibility criteria included randomized controlled trials (RCTs) and non-RCT studies (i.e., non-randomized clinical trials, observational studies) of patients with knee OA receiving treatment with either one injection of hylan G-F 20 (Synvisc-One®, Genzyme Biosurgery, Ridgefield, NJ, USA) or three injections of hylan G-F 20 (Synvisc®, Genzyme Biosurgery, Ridgefield, NJ, USA). We required 1-year posttreatment assessment of one of the following: visual analogue scale (VAS) pain, Western Ontario and McMaster Universities Arthritis Index (WOMAC), 36-item short form survey (SF-36), adverse events (AEs), drug discontinuation due to AEs, or study withdrawal due to AEs.

The following databases were searched from January 1, 1995, to October 18, 2018: PubMed, Embase, and the Cochrane Central Register of Controlled Trials (Supplementary Material Table S1-S3). An update to the search was conducted covering the gap from October 18, 2018, to August 17, 2020, in order to capture any new eligible studies that may have been published from the initial search date.

To ensure that all relevant studies were included, a hand-search of the bibliography of key included studies and previously published systematic reviews was performed. Conference proceedings were also included via Embase. Publications were restricted to those published in the English language and those studying human subjects. Title and abstract screening and subsequent full-text screening were done independently and in duplicate by two reviewers. Discrepancies during the screening and extraction process were reconciled through discussion or resolved by a third reviewer.

### Outcome measures

Efficacy and safety outcomes of interest reported at 1 year were extracted. Primary efficacy outcomes were proportions of patients with any response, mean, median, change, and percent change in WOMAC pain, physical function, and stiffness. Secondary outcomes were VAS pain and SF-36. Safety outcomes were proportions of patients who experienced AEs, serious/severe AEs, treatment-related AEs, target knee AEs, and drug discontinuation/study withdrawal due to AEs. All safety outcomes were extracted as classified by the authors of the studies. Target knee AEs were defined as the total of any AEs reported specifically for the knee receiving treatment.

### Risk of bias assessment

Study quality assessments of included studies were conducted using the Cochrane Risk of Bias Tool [27] for RCTs and the Newcastle-Ottawa Scale for non-RCTs [28]. Discrepancies in ratings of study quality were reconciled through discussion or resolved by a third reviewer. We used the GRADE (Grading of Recommendations Assessment, Development, and Evaluation) method to appraise the quality of evidence [29].

### Statistical analysis

Outcomes were measured using various scales, so results were converted to standardized mean change (SMCC). Scores were standardized by dividing the average change by the standard deviation (SD). When the mean change and SD were not available, they were calculated from the group level information, and correlation was assumed to be 0.5. When the mean change and SD were unavailable, but percent change of mean was reported, the final mean and mean change were calculated from the percent change. The formula used for this purpose was:

$$ \mathrm{Percentage}\ \mathrm{Change}=\frac{End\  Point- Baseline}{Baseline}\ast 100 $$

Imputation was done to fill in missing SD with consideration of the range value. Each component of the scale was analyzed on its own. The strict “Pain” outcome was used when available; however, if the study only reported a sub-scale of pain (e.g., WOMAC walking pain), it was used instead. A random-effects model was used to derive the effects of outcomes. For continuous outcomes of WOMAC, VAS pain, and SF-36, SMCC was used for effect sizes. For dichotomous outcomes of AEs, drug discontinuation due to AEs, and study withdrawal due to AEs, composite of logit transformed proportion (log odds) was derived, and transformation inverse of logit transformation was used. The alpha value of 0.05 was used to establish statistical significance, and *I*^2^ was used to assess heterogeneity across studies. All analyses were performed with R version 3.0.3 (http://www.r-project.org/) using R package “metafor.”

## Results

### Search results

The search retrieved a total of 404 records after duplicates were removed, 400 of which were from database searches and four additional studies from hand-searching the reference list of relevant publications. After 260 were excluded during title and abstract screening for failing to meet PICO criteria (e.g., due to wrong study design, wrong population), 144 studies underwent full-text screening. During full-text screening, 121 additional studies did not meet the PICO criteria, and 24 were included for data extraction. Of the 24 studies that were extracted, 18 were included in the meta-analysis. The PRISMA diagram can be found in the Supplementary Material Fig. S1. The gap search from October 2018 to August 2020 identified 54 new records, after title and abstract screening 49 records were excluded (mainly due to wrong study design) leaving five records for full-text review. None of these five additional studies were eligible for inclusion (two were excluded for wrong study design, two for outcomes of interest not reported, and one for intervention due to evaluating a mixed group of different IAHAs with no stratification of results).

### Study and patient characteristics

Supplementary Material Table S4 shows the study and patient characteristics extracted from the included studies. Of the 24 included studies, there were 13 RCTs [22, 30–42] representing 10 unique samples, six non-randomized clinical trials [21, 43–47], four non-comparative observational studies [48–51], and one case series [52]. Seven studies reported on single hylan G-F 20 injection [21, 30, 48–52] and 17 reported on 1–3 weekly injections of hylan G-F 20 [22, 31–47]. Other interventions consisted of appropriate care, arthroscopy, Artzal®, Durolane®, knee lavage, Hyalgan®, Orthrovisc®, Ostenil®, physical therapy agents, placebo, platelet-rich plasma, standard care, and Suprahyal®/Adant®. The number of patients in each treatment arm ranged from 10 [42] to 451 [48]. The ages ranged from a mean of 53.1 years [41] to 72 years [37]. The included studies consisted of a mixture of patients with mild to severe knee OA, Kellgren-Lawrence grade 0–4, and Ahlbäck classification grade 1–3.

### Outcomes

The Supplementary Material Table S5 and Table S6 include the extracted results of the 1-year efficacy and safety outcomes, respectively. Some studies assessed WOMAC using the Likert scale, while others used the VAS.

### Study quality

The study quality of 10 trials (reflecting 13 publications) were assessed using the Cochrane Risk of Bias Tool (Supplementary Material Table S7) [22, 30, 31, 34–39, 41, 42]. Bellamy et al. [32], Bellamy et al. [33], and Raynauld et al. [40] were not assessed as they are post hoc analyses of the same sample reported on in Raynauld et al. [22]. The majority of the studies were assessed as low or unclear risk for most categories except for attrition bias. The risk of attrition bias was high in eight trials due to high rates of dropouts, differential rates of dropouts between groups, or missing data being imputed using inappropriate methods. Additional details on the quality assessment of trials can be found in the Supplementary Material Table S8 and Fig. S2.

The quality of 10 non-RCT studies (reflecting 10 publications) was assessed with the Newcastle-Ottawa Scale (Supplementary Material Table S9). Overall, studies were mainly rated as moderate quality. Nine studies were rated as moderate quality [43–51], and one study was rated as high quality [21]. Additional details on the Newcastle-Ottawa Scale assessments can be found in the Supplementary Material Table S10. Boutefnouchet et al. [52] is a case series and was not assessed using the Newcastle-Ottawa Scale as the scale was designed for cohort and case-control studies.

### Treatment schedules: courses of 1–3 weekly injections of hylan G-F 20 injection and concomitant medication

Studies varied in permitted concomitant therapies, as well as in the administration of 1–3 weekly injections of hylan G-F 20 versus single hylan G-F 20 injection. Seventeen studies allowed additional courses of hylan G-F or concomitant medications for pain [21, 22, 32, 33, 35, 37–44, 47–50, 52]. One study specified that no patients receive more than one course of injections [45], and another study required patients to discontinue the use of nonsteroidal anti-inflammatory drugs and analgesics [51]. Although the original formulation of hylan G-F 20 is administered in three injections, patients in one study received a total of four injections [31]. The remaining four studies did not specify additional injections or concomitant medications [30, 34, 36, 51]. Only Raynauld et al. and Waddell et al. [40, 47] reported data separately for groups of patients receiving a single-course or repeat-course of hylan G-F 20. In Raynauld et al., the hylan G-F 20 1–3 weekly injections single-course and repeat-course groups were not statistically significantly different from one another, and both groups showed statistically significant improvement in WOMAC pain over the appropriate care group [40]. Waddell et al. reported significant improvement in total WOMAC, WOMAC pain while walking, and WOMAC physical function, and the treatments were generally well tolerated [47].

### Meta-analytic results

#### WOMAC total

**Fig. 1:**
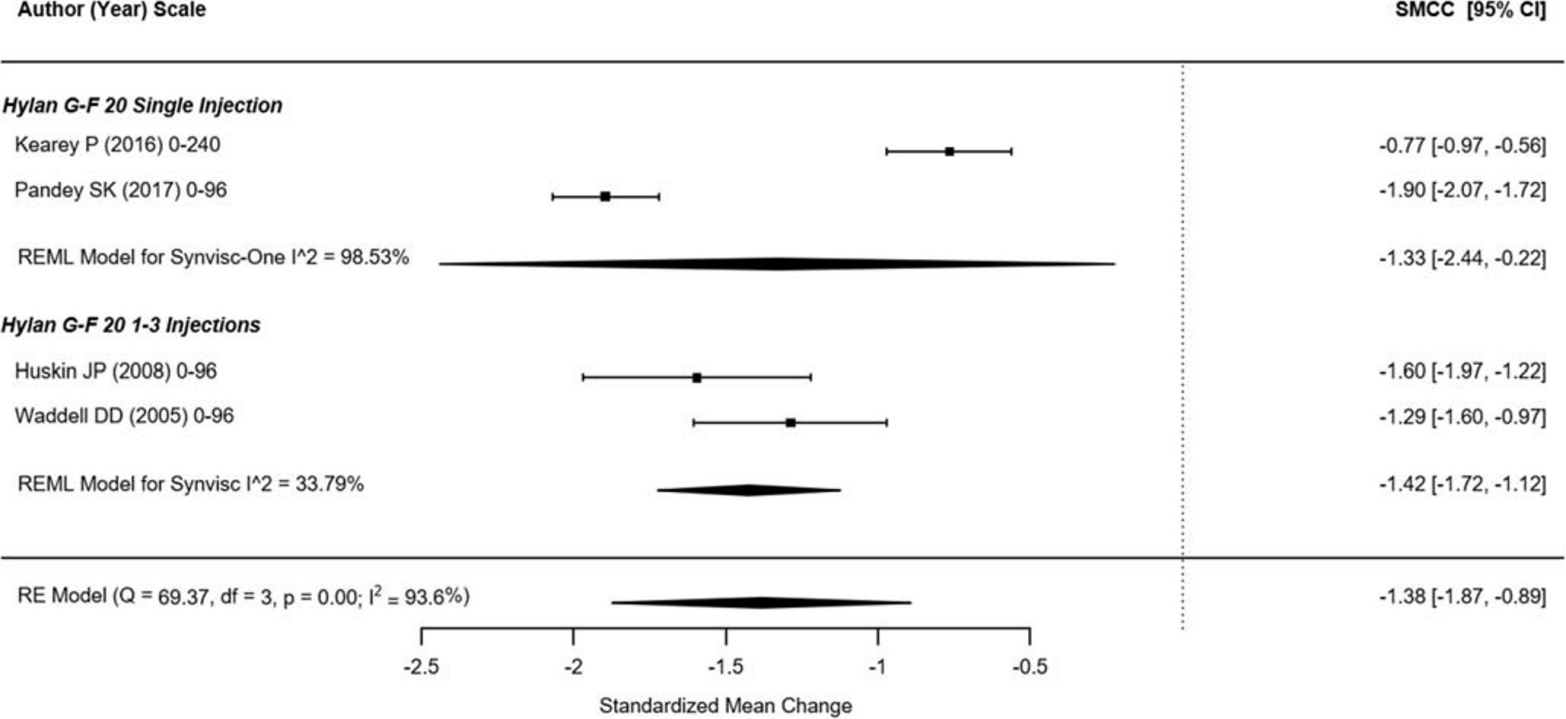
WOMAC total (non-RCTs)

Four non-randomized studies (representing 603 participants) which reported the Total WOMAC outcome were included in this analysis (Fig. 1) [44, 47, 49, 50]. The SMCC (95% confidence interval [CI]) of single hylan G-F 20 injection and hylan G-F 20 1–3 weekly injection groups were − 1.33 (95% CI: − 2.44, − 0.22) and − 1.42 (95% CI: − 1.72, − 1.12), respectively. The overall SMCC for hylan G-F 20 among these non-randomized studies showed statistically significant improvement of − 1.38 (95% CI: − 1.87, − 0.89; *p* < .0001). For comparison purposes, the SMCC for hylan G-F 20 provided by the sole RCT of 10 participants was − 2.28 (95% CI: − 3.46, − 1.10), a significant improvement (*p* < .001) [42]. The quality of evidence for WOMAC total score was rated as low (downgraded due to imprecision and risk of bias)

#### WOMAC pain

**Fig. 2:**
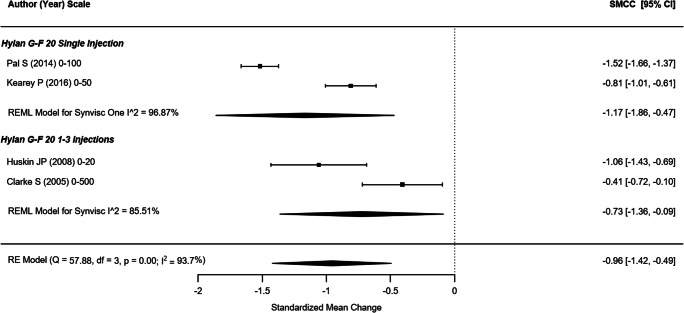
WOMAC pain (non-RCTs)

**Fig. 3:**
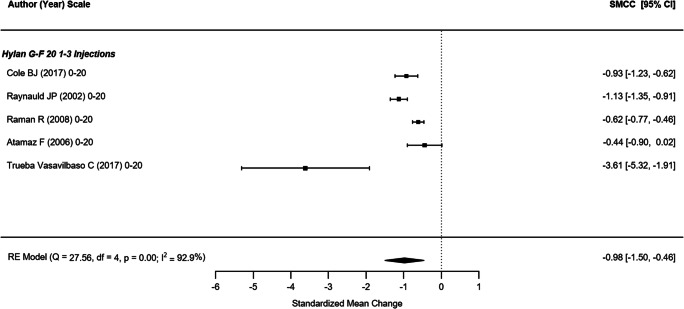
WOMAC pain (RCTs)

Four non-randomized studies (representing 709 participants) that reported the WOMAC pain outcome were included in this analysis (Fig. 2) [21, 43, 44, 49]. The SMCC of single hylan G-F 20 injection and hylan G-F 20 1–3 weekly injection groups were − 1.17 (95% CI: − 1.86, −0.47) and − 0.73 (95% CI: − 1.36, − 0.09), respectively. The overall SMCC for hylan G-F 20 showed a reduction in pain, SMCC = − 0.96 (95% CI: − 1.42, − 0.49). All effects were statistically significant (*p* < .001). Among the five RCTs that reported WOMAC pain scores across 415 participants (Fig. 3) [22, 31, 34, 39, 42], the SMCC for hylan G-F 20 was − 0.98 (95% CI: − 1.50, − 0.46), a significant reduction (*p* < .001). The quality of evidence for pain (as measured by either WOMAC or VAS) was rated as moderate (downgraded due to inconsistency).

#### WOMAC physical function

**Fig. 4:**
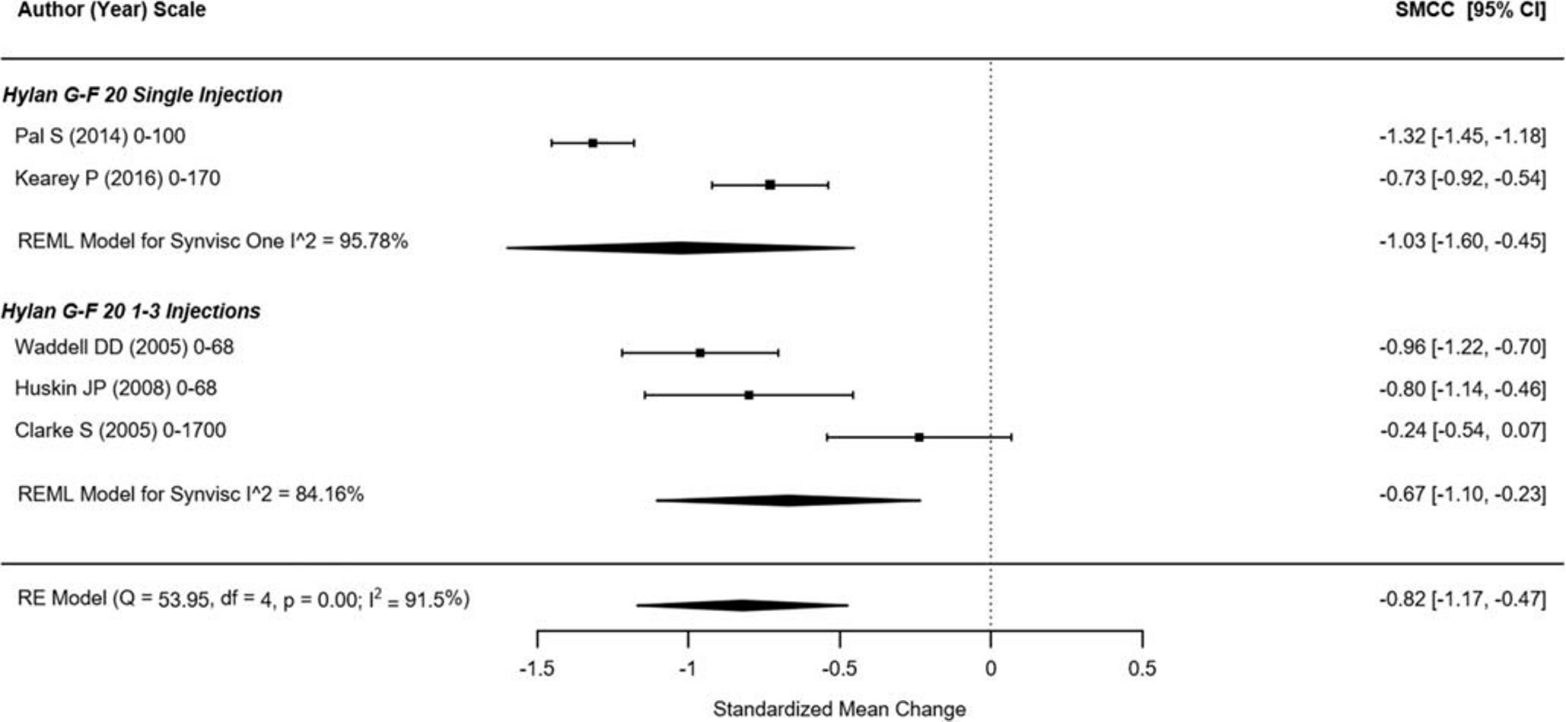
WOMAC physical function (non-RCTs)

**Fig. 5:**
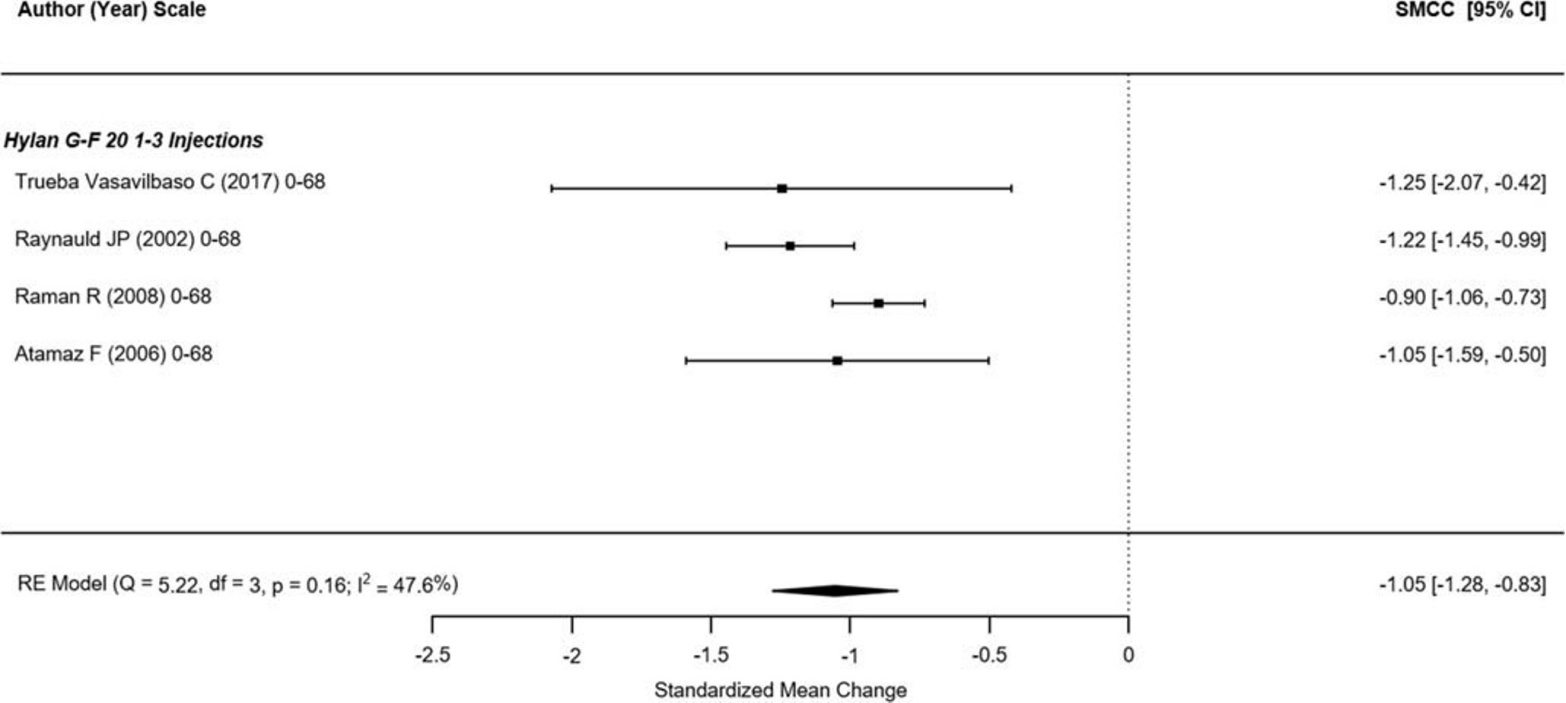
WOMAC physical function (RCTs)

Five non-randomized studies (representing 709 participants) which reported the WOMAC physical function outcome were included in this analysis (Fig. 4) [21, 43, 44, 47, 49]. The SMCC of single hylan G-F 20 injection and hylan G-F 20 1–3 weekly injection groups were − 1.03 (95% CI: − 1.60, − 0.45) and − 0.67 (95% CI: − 1.10, − 0.23), respectively. The overall SMCC for hylan G-F 20 showed improvement in physical function, SMCC = − 0.82 (95% CI: − 1.17, − 0.47; *p* < .0001). Among the four RCTs that reported WOMAC physical function scores across 356 participants (Fig. 5), the SMCC for hylan G-F 20 was − 1.05 (95% CI: − 1.28, − 0.83), a significant improvement (*p* < .001) [22, 31, 39, 42]. The quality of evidence for physical function was rated as high.

#### WOMAC stiffness

Four non-randomized studies (representing 624 participants) which reported the WOMAC stiffness outcome were included in this analysis (Supplementary Material Fig. S3) [21, 43, 44, 49]. The SMCC of the single hylan G-F 20 injection and hylan G-F 20 1–3 weekly injection groups were − 0.98 (95% CI: − 1.36, − 0.59) and − 0.93 (95% CI: − 2.07, 0.22), respectively. The overall SMCC for hylan G-F 20 showed a reduction of − 0.95 (95% CI: − 1.42, − 0.47; *p* < .0001). Among the two RCTs that reported WOMAC stiffness scores across 137 participants (Supplementary Material Fig. S4), the SMCC for hylan G-F 20 was − 1.07 (95% CI: − 1.28, − 0.86), a significant reduction (*p* < .001) [22, 42]. The quality of evidence for stiffness was rated as high.

#### VAS pain

Two non-randomized studies (representing 132 participants) that reported the VAS pain outcome were included in this analysis (Supplementary Material Fig. S5) [46, 51]. The SMCC of single hylan G-F 20 injection and hylan G-F 20 1–3 weekly injection groups were − 0.54 (95% CI: − 0.75, − 0.32) and − 4.39 (95% CI: − 5.44, − 3.34), respectively. The overall SMCC for hylan G-F 20 showed a reduction in pain of − 2.42 (95% CI: − 6.20, 1.35), which was not significant (*p* = .21) due to the extreme heterogeneity between the two injection schedules (*I*^2^ = 98%). Among the three RCTs that reported VAS pain scores across 278 participants (Supplementary Material Fig. S6), the SMCC for hylan G-F 20 was − 1.58 (95% CI: − 2.97, − 0.19), a significant reduction (*p* = .025) [31, 34, 39]. The quality of evidence has already been described above (see WOMAC pain).

#### SF-36 mental and physical component summary

One non-randomized study of 107 participants reported the SF-36 mental and physical component summary outcomes, showing a SMCC of 0.13 (95% CI: − 0.06, 0.32) for both components [49]. For the sole RCT, the reported SF-36 mental and physical component scores improved among 127 participants: the SMCC was 0.27 (95% CI: 0.10, 0.45, *p* = .002) and 0.50 (95% CI: 0.31, 0.68, *p* < .001), respectively, for the mental and physical components [22]. The quality of evidence for this outcome was rated as low (downgraded due to inconsistency and imprecision).

### Overall adverse events

Single hylan G-F 20 injection had an average AE rate of 11% (95% CI: 3–38%) across three non-randomized studies (Supplementary Material Fig. S7) [48, 49, 51]. Hylan G-F 20 1–3 weekly injection had an average AE rate of 85% (95% CI: 26–99%) across two RCTs (Supplementary Material Fig. S8), though in these studies the rate of AEs in the treatment group was only 1–6% higher than the comparison group [22, 41]. That is, Raynauld et al. reported AEs in 96% of hylan G-F 20 1–3 weekly injections and 90% in the appropriate care group [22]. Rolf et al. reported AEs in 59% of hylan G-F 20 1–3 weekly injections, 60% of the hyaluronan (Artzal®) group, and 60% in the placebo group [41]. An exhaustive list of the types of AEs reported across included studies can be found in Supplementary Table S11.

### Serious adverse events

Few studies reported on serious adverse events. Single hylan G-F 20 injection had an average serious AE rate of 1% (95% CI: 0–7%) across two non-randomized studies (Supplementary Material Fig. S9) [21, 48]. Hylan G-F 20 1–3 weekly injections had an average serious AE rate of 0% (95% CI: 0–2%) across two RCTs (Supplementary Material Fig. S10) [22, 39].

### Treatment-related adverse events

Few studies distinguished and reported on treatment-related AEs. Single hylan G-F 20 injections had an average treatment-related AE rate of 2% (95% CI: 1–4%) across two non-randomized studies (Supplementary Material Fig. S11) [21, 52]. Hylan G-F 20 1–3 weekly injections had an average treatment-related AE rate of 8% (95% CI: 1–35%) across three RCTs (Supplementary Material Fig. S12) [36, 39, 42]. This latter number was driven up by Raman et al., who reported a treatment-related AE rate of 20% among the hylan G-F 20 group and 16% among the sodium hyaluronate group.

### Target knee adverse events

Two non-randomized studies reported on target knee AEs. Single hylan G-F 20 injections had a target knee AE rate of 6% in one study [21]. Hylan G-F 20 1–3 weekly injections had a target knee adverse event rate of 37% in another study [43], with authors noting “the relationship of these events to the administration of hylan G-F 20 is not clear and includes 2 patients who complained of knee pain following falls.” No RCTs reported on target knee adverse events.

### Drug discontinuation due to adverse events

Two multiple injection studies reported on drug discontinuation due to adverse events. The average drug discontinuation due to AEs in hylan G-F 20 1–3 weekly injections was 1% in an RCT by Karlsson et al. and 5% in a non-randomized study by Clarke et al. [37, 43].

## Discussion

Considering the lack of consensus among current meta-analyses [14] and practice guidelines involving the use of HA for the treatment of knee OA [15, 16], we conducted a focused meta-analysis on the safety and efficacy of one specific formulation (hylan G-F 20, administered either 1–3 times weekly or a single injection). We chose the HA formulation mainly because it is among the most frequently studied, thereby providing the most data for a focused meta-analysis. As some studies have reported that the treatment efficacy for some HA formulations is short-lived, we restricted our search to studies that included outcomes 12 months posttreatment.

Consistent with previous meta-analytic findings (based largely on briefer follow-up periods), hylan G-F 20 injections showed statistically significant improvements on pain, physical function, and joint stiffness. The current findings advance the field by demonstrating that not only do these significant effects persist a year later, but they are also on average a full standard deviation in magnitude. Some recent literature includes the conclusion that all HA formulations show efficacy effects small enough to merit their discontinuation as a treatment strategy [12]. The current results show that not all HA formulations necessarily merit such a dismissal, as a full standard deviation change in pain, stiffness, and physical function may be clinically meaningful for patients.

Previous reviews of this topic have grouped all HA formulations [12, 13]. This approach increases statistical power, but risks combining treatments with heterogenous efficacy or risk. Our focus on the 1-year efficacy and safety of single and 1–3 weekly injections of hylan G-F 20 reveals several new insights. First, the efficacy estimates are higher than previous meta-analyses which combine hylan G-F 20 with other HA formulations [12]. Second, the rates of adverse events (overall, treatment-related, serious) are lower than previous meta-analyses that included various HA formulations [13]. HA formulations vary in their molecular weight, volume, concentration, and other factors; attempting to combine across these heterogenous treatments in meta-analyses may contribute to the inconsistency noted in this area of research. Additional focused meta-analyses which do not combine HA formulations may benefit this field given the lack of consensus among current meta-analyses and even practice guidelines involving the use of HA as a treatment for knee OA [15, 16].

This meta-analysis has several limitations. Due to the nature of OA treatment, most studies allowed additional concomitant medication or additional courses of hylan G-F 20. Studies that did not prohibit additional injections or concomitant treatments may have confounded the results. The potential confounding effects should be further studied, but such investigations were not feasible here given the limitations of the data. Some of the meta-analyses had high levels of heterogeneity (*I*^2^ > 75%), potentially caused by variation in unmeasured patient characteristics. Attrition was high in the majority of these studies, which reduces the generalizability of these trials to all patients with knee OA who receive HA injections. Non-English studies were excluded, which could have introduced bias [53]. Finally, our focus was on a specific set of outcomes, and other outcomes could produce a different pattern of results.

In conclusion, single and 1–3 weekly injections of hylan G-F 20 significantly improved WOMAC (total, pain, physical function, and stiffness), VAS pain, SF-36 MCS, and SF-36 PCS at 1 year after first injection. Single and 1–3 weekly injections of hylan G-F 20 are efficacious and generally safe for long-term use.

## Supplementary Information

ESM 1 (PDF 792 kb)

## Data Availability

Not applicable.
