# Supplementary material for: One-year efficacy and safety of single or one to three weekly injections of hylan G-F 20 for knee osteoarthritis: a systematic literature review and meta-analysis
Source: Clin Rheumatol. 2020 Oct 27;40(6):2133–42. doi: 10.1007/s10067-020-05477-7 (PMC8121739; doi:10.1007/s10067-020-05477-7)
Supplement: Supplementary file 1 — (PDF 792 kb) [file 10067_2020_5477_MOESM1_ESM.pdf]

One-Year Efficacy and Safety of Single or One to Three Weekly Injections of Hylan G-F 20 for Knee Osteoarthritis: A Systematic Literature Review and Meta-Analysis

Orazio De Lucia, MD;[1] Joerg Jerosch, MD;[2] Sophie Yoon, MPH;[3] Tobias Sayre, MS;[3] Wilson Ngai, MSc;[4] Georgios Filippou, MD[5]

[1] Clinical Rheumatology Unit, Department of Rheumatology and Medical Sciences, ASST Centro Traumatologico Ortopedico G. Pini-CTO, Milan, Italy

[2] Orthopedic Department, Johanna Étienne Hospital, Am Hasenberg 46, 41462 Neuss, Germany

[3] Doctor Evidence, Santa Monica, USA

[4] Global Medical Affairs, Sanofi, Bridgewater, NJ, USA

[5] ASST Fatebenefratelli, University Hospital Sacco, Rheumatology Unit, Milan - Italy

Corresponding Author: Georgios Filippou

Via Giovanni Battista Grassi, 74, 20157 Milano MI, Italy

gf.filippou@gmail.com

Journal: *Clinical Rheumatology*

Supplemental Material

Table S0 PICO Eligibility Criteria

PICO	Inclusion Criteria
**Participants**	• Persons with knee osteoarthritis
**Interventions**	• Hylan G-F 20 (single and 1-3 weekly injections)
**Comparators**	• Any<br>• None
**Outcomes**	• Response Rate<br>• VAS Pain<br>• WOMAC<br>• SF-36<br>• Adverse Events (Total, Serious, Severe, Treatment-Related, Target Knee)<br>• Drug Discontinuation due to Adverse Events<br>• Study Withdrawal due to Adverse Events
**Timing**	• 1 year

**Setting**	• Any
**Study Design**	• Randomized Controlled Trials<br>• Non-Randomized Clinical Trials<br>• Observational Studies

Table S1 MEDLINE (via PubMed) Search Strategy

Searched: October 18, 2018

Set #	Searches	Results
1	"Osteoarthritis, Knee"[Mesh]	16744
2	"Knee Joint"[Mesh] OR "Knee"[Mesh] OR (Femoropatellar[tiab] OR femoro-patellar[tiab] OR knee[tiab] OR knees[tiab] OR kneecap[tiab] OR kneecaps[tiab] OR knee-cap[tiab] OR knee-caps[tiab] OR patella[tiab] OR patellas[tiab] OR patellar[tiab] OR patellofemoral[tiab] OR patello-femoral[tiab] OR tibiofemoral[tiab] OR tibio-femoral[tiab] OR tibiofibular[tiab] OR tibio-fibular[tiab])	155563
3	"Osteoarthritis"[Mesh:NoExp] OR (arthriti*[tiab] OR degenerative arthro*[tiab] OR degenerative joint[tiab] OR degenerative joints[tiab] OR gonarthriti*[tiab] OR gonarthro*[tiab] OR osteoarthriti* OR osteo-arthriti* OR osteoarthros*[tiab] OR osteo-arthros*[tiab] OR OA[tiab])	247612
4	2 AND 3	40750
5	1 OR 4	42054
6	"hylan" [Supplementary Concept] OR synvisc[tiab] OR hylan g f 20[tiab] OR hylan g-f 20[tiab] OR hylan gf-20[tiab] OR hylan gf 20[tiab]	297
7	Synvisc*[tiab] OR Synvisc-One[tiab]	108
8	6 OR 7	297
9	5 AND 8	205
10	English[Language]	24302437
11	9 AND 10	195
12	"1995/01/01"[PDAT] : "3000/12/31"[PDAT]	17824460
13	11 AND 12	194

Table S2 Embase (via OVID) Search Strategy

Searched: October 18, 2018

Embase Segment Used: 1974 to October 17, 2018

Set #	Searches	Results
1	exp knee osteoarthritis/	27443
2	exp knee/	56537
3	exp patella/	8294
4	exp patellofemoral joint/	3825
5	(Femoropatellar or femoro-patellar or knee or knees or kneecap or kneecaps or knee-cap or knee-caps or patella or patellas or patellar or patellofemoral or patello-femoral or tibiofemoral or tibio-femoral or tibiofibular or tibio-fibular).ti,ab,kw.	178810
6	or/2-5	185851
7	exp osteoarthritis/	112888
8	(arthriti$ or degenerative arthriti$ or degenerative arthro$ or degenerative joint or degenerative joints or gonarthriti$ or gonarthro$ or osteoarthriti$ or osteo-arthriti$ or osteoarthros$ or osteo-arthros$ or OA).ti,ab,kw.	321434
9	7 or 8	357680
10	6 and 9	57281
11	1 or 10	60727
12	(Synvisc or Synvisc$ or Synvisc-One or Synvisc One or Synvisc-1 or Synvisc 1).mp.	464
13	(hylan g f 20 or hylan g-f 20 or hylan gf-20 or hylan gf 20).mp.	315
14	12 or 13	599
15	11 and 14	414
16	(article or article in press or conference abstract or conference paper).pt.	23978506
17	15 and 16	307
18	English.lg.	26457670
19	17 and 18	290
20	limit 19 to yr="1995 -Current"	288

Table S3 Cochrane Central Database of Controlled Trials (via OVID) Search Strategy

Searched: October 18, 2018

**Set #**	**Searches**	**Results**
1	exp Osteoarthritis, Knee/	3216
2	exp Knee/ or exp Knee Joint/ or exp Patella/ or exp patellofemoral joint/	3639
3	knee$.ti,ab,kw,hw,sh.	20019
4	(Femoropatellar or femoro-patellar or knee or knees or kneecap or kneecaps or knee-cap or knee-caps or patella or patellas or patellar or patellofemoral or patello-femoral or tibiofemoral or tibio-femoral or tibiofibular or tibio-fibular).ti,ab,kw,hw,sh.	20461
5	or/2-4	20563
6	exp osteoarthritis/	5941
7	(arthriti$ or degenerative arthriti$ or degenerative arthro$ or degenerative joint or degenerative joints or gonarthriti$ or gonarthro$ or osteoarthriti$ or osteo-arthriti$ or osteoarthros$ or osteo-arthros$ or OA).ti,ab,kw,hw,sh.	25241
8	6 or 7	25241
9	5 and 8	8283
10	1 or 9	8283
11	(synvisc or synvisc one).ti,ab,kw,hw,sh.	53
12	(hylan g f 20 or hylan g-f 20 or hylan gf-20 or hylan gf 20).ti,ab,kw,hw,sh.	78
13	11 or 12	105
14	10 and 13	94
15	English.lg.	807813
16	14 and 15	69
17	limit 16 to yr="1995-2018"	69

Table S4 Study and patient characteristics

Author and Year	Study Design	Interventions	Number of Patients	Mean Age	OA Grade
**Acharya KKV 2013**[27]	RCT	Hylan G-F Single injection	41	NR	Osteoarthritis, Knee, Mild to Moderate - 82 (100%)<br>Kellgren-Lawrence Classification, Grade 1-3 - 82 (100%)
		Arthroscopy	41		
**Atamaz F 2006**[28]	RCT	Hylan G-F 1-3 weekly injections	20	60.4 yr (SD ± 9.3)	Kellgren-Lawrence Classification, Grade 2-3 - 82 (100%)
		Sodium hyaluronate + sodium chloride (Orthovisc®)	20	62.4 yr (SD ± 9)	
		Physical Therapy Agents	42	58.7 yr (SD ± 8.3)	
**Bellamy N 2005a**[29]	RCT	Hylan G-F 1-3 weekly injections	127	62.6 yr (SD ± 9.4)	Osteoarthritis, Knee, Mild to Moderate - 127 (100%)<br>Kellgren-Lawrence Classification, Grade, Not Reported - 0 (0%)<br>Kellgren-Lawrence Classification, Grade 0 - 4 (3%)<br>Kellgren-Lawrence Classification, Grade 1 - 17 (13%)<br>Kellgren-Lawrence Classification, Grade 2 - 32 (25%)<br>Kellgren-Lawrence Classification, Grade 3 - 49 (39%)<br>Kellgren-Lawrence Classification, Grade 4 - 25 (20%)
		Appropriate Care	128	63.5 yr (SD ± 10.5)	Osteoarthritis, Knee, Mild to Moderate - 128 (100%)<br>Kellgren-Lawrence Classification, Grade, Not Reported - 1 (1%)<br>Kellgren-Lawrence Classification, Grade 0 - 4 (3%)<br>Kellgren-Lawrence Classification, Grade 1 - 11 (9%)<br>Kellgren-Lawrence Classification, Grade 2 - 33 (26%)<br>Kellgren-Lawrence Classification, Grade 3 - 37 (29%)<br>Kellgren-Lawrence Classification, Grade 4 - 42 (33%)
**Bellamy N 2005b**[30]	RCT	Hylan G-F 1-3 weekly injections	127	62.6 yr (SD ± 9.4)	Osteoarthritis, Knee, Mild to Moderate - 127 (100%)<br>Kellgren-Lawrence Classification, Grade, Not Reported - 0 (0%)<br>Kellgren-Lawrence Classification, Grade 0 - 4 (3%)

Author and Year	Study Design	Interventions	Number of Patients	Mean Age	OA Grade
					Kellgren-Lawrence Classification, Grade 1 - 17 (13.39%)<br><br>Kellgren-Lawrence Classification, Grade 2 - 32 (25%)<br><br>Kellgren-Lawrence Classification, Grade 3 - 49 (39%)<br><br>Kellgren-Lawrence Classification, Grade 4 - 25 (20%)
		Appropriate Care	128	63.5 yr (SD ± 10.5)	Osteoarthritis, Knee, Mild to Moderate - 128 (100%)<br><br>Kellgren-Lawrence Classification, Grade, Not Reported - 1 (1%)<br><br>Kellgren-Lawrence Classification, Grade 0 - 4 (3%)<br><br>Kellgren-Lawrence Classification, Grade 1 - 11 (9%)<br><br>Kellgren-Lawrence Classification, Grade 2 - 33 (26%)<br><br>Kellgren-Lawrence Classification, Grade 3 - 37 (29%)<br><br>Kellgren-Lawrence Classification, Grade 4 - 42 (33%)
**Boutefnouchet T 2017**[49]	Case Series	Hylan G-F Single injection	77	58 yr (SD ± 11.9, Total: 32 – 88, 95% CI: 51.5 – 73.2)	NR
**Clarke S 2005**[40]	Non-Controlled Clinical Trial	Hylan G-F 1-3 weekly injections	43	56 yr (SD ± 10.6, Total: 36 – 82, 95% CI: 52.4 – 59)	NR
**Cole BJ 2017**[31]	RCT	Hylan G-F 1-3 weekly injections	59	56.8 yr (SD ± 10.5)	Osteoarthritis, Knee, Mild to Moderate - 59 (100%)<br><br>Kellgren-Lawrence Classification, Grade 1-4 - 59 (100%)<br><br>Kellgren-Lawrence Classification, Grade, Unknown - 1 (2%)<br><br>Kellgren-Lawrence Classification, Grade 1 - 0 (0%)<br><br>Kellgren-Lawrence Classification, Grade 2 - 27 (54%)<br><br>Kellgren-Lawrence Classification, Grade 3 - 22 (44%)
		Platelet-Rich Plasma	52	55.9 yr (SD ± 10.4)	Osteoarthritis, Knee, Mild to Moderate - 52 (100%)<br><br>Kellgren-Lawrence Classification, Grade 1-4 - 52 (100%)<br><br>Kellgren-Lawrence Classification, Grade, Unknown - 0 (0%)<br><br>Kellgren-Lawrence Classification, Grade 1 - 3 (6.12%)<br><br>Kellgren-Lawrence Classification, Grade 2 - 26 (53.06%)

Author and Year	Study Design	Interventions	Number of Patients	Mean Age	OA Grade
					Kellgren-Lawrence Classification, Grade 3 - 22 (44%) - 20 (40.82%)
**Daniel D 2011**[45]	Observational Non-Comparative	Hylan G-F Single injection	451		Kellgren-Lawrence Classification, Grade 1-3 - 451 (100%)<br>Kellgren-Lawrence Classification, Grade 1 - 42 (9.3%)<br>Kellgren-Lawrence Classification, Grade 2 - 175 (38.8%)<br>Kellgren-Lawrence Classification, Grade 3 - 234 (51.9%)
**Huskin JP 2008**[41]	Non-Controlled Clinical Trial	Hylan G-F 1-3 weekly injections	62	55.4 yr (SD ± 11.35)	Osteoarthritis, Knee, Mild to Moderate - 62 (100%)<br>Kellgren-Lawrence Classification, Grade 1-2 -<br>Kellgren-Lawrence Classification, Medial, Grade 1 - 36 (58%)<br>Kellgren-Lawrence Classification, Medial, Grade 2 - 25 (40%)<br>Kellgren-Lawrence Classification, Medial, Grade 3 - 1 (2%)<br>Kellgren-Lawrence Classification, Lateral, Grade 1 - 50 (80%)<br>Kellgren-Lawrence Classification, Lateral, Grade 2 - 12 (20%)<br>Kellgren-Lawrence Classification, Patellofemoral, Grade 1 - 52 (84%)<br>Kellgren-Lawrence Classification, Patellofemoral, Grade 2 - 10 (16%)
**Juni P 2007**[32]	RCT	Hylan G-F 1-3 weekly injections	222	63.3 yr (SD ± 12.3)	Osteoarthritis, Knee, Radiographic, Moderate - 126 (57%)<br>Osteoarthritis, Knee, Radiographic, Severe - 43 (19%)<br>Osteoarthritis, Knee, Clinical, Moderate - 42 (19%)<br>Osteoarthritis, Knee, Clinical, Severe - 143 (64%)<br>Kellgren-Lawrence Classification, Grade 2-4 - 222 (100%)
		Medium Molecular Weight Hyaluronic Acid (Orthovisc®)	219	63.5 yr (SD ± 11.1)	Osteoarthritis, Knee, Radiographic, Moderate - 127 (58%)<br>Osteoarthritis, Knee, Radiographic, Severe - 48 (22%)<br>Osteoarthritis, Knee, Clinical, Moderate - 39 (18%)<br>Osteoarthritis, Knee, Clinical, Severe - 145 (66%)<br>Kellgren-Lawrence Classification, Grade 2-4 - 219 (100%)

Author and Year	Study Design	Interventions	Number of Patients	Mean Age	OA Grade
		Non–cross-linked low molecular weight Hyaluronic Acid (Ostenil®)	219	63.3 yr (SD ± 11.5)	Osteoarthritis, Knee, Radiographic, Moderate - 131 (60%)<br>Osteoarthritis, Knee, Radiographic, Severe - 40 (18%)<br>Osteoarthritis, Knee, Clinical, Moderate - 52 (24%)<br>Osteoarthritis, Knee, Clinical, Severe - 138 (63%)<br>Kellgren-Lawrence Classification, Grade 2-4 - 219 (100%)
**Karatosun V 2005[33]**	RCT	Hylan G-F 1-3 weekly injections	46	60.5 yr (SD ± 9.5)	Osteoarthritis, Knee, Severe - 46 (100%)<br>Kellgren-Lawrence Classification, Grade 3 - 46 (100%)<br>Kellgren-Lawrence Classification, Grade 4 - 0 (0%)
		Low-Molecular Weight Hyaluronic acid - 3 injections (Orthovisc®)	46	60.6 yr (SD ± 9.6)	Osteoarthritis, Knee, Severe - 46 (100%)<br>Kellgren-Lawrence Classification, Grade 3 - 46 (100%)<br>Kellgren-Lawrence Classification, Grade 4 - 0 (0%)
**Karlsson J 2002[34]**	RCT	Hylan G-F 1-3 weekly injections	88	70 yr (SD ± 7)	Ahlbäck Classification, Grade 1-2 - 88 (100%)<br>Ahlbäck Classification, Grade 3-5 - 0 (0%)<br>Ahlback Classification, Grade 1- 52 (61%)<br>Ahlback Classification, Grade 2 - 34 (39%)
		1% hyaluronan (Artzal®) – 3 injections per week	92	72 yr (SD ± 7)	Ahlbäck Classification, Grade 1-2 - 92 (100%)<br>Ahlbäck Classification, Grade 3-5 - 0 (0%)<br>Ahlback Classification, Grade 1 - 54 (60%)<br>Ahlback Classification, Grade 2 - 36 (40%)
		Placebo	66	71 yr (SD ± 6)	Ahlbäck Classification, Grade 1-2 - 66 (100%)<br>Ahlbäck Classification, Grade 3-5 - 0 (0%)<br>Ahlback Classification, Grade 1 - 38 (58%)<br>Ahlback Classification, Grade 2 - 28 (42%)
**Kearey P 2016[46]**	Observational Non-Comparative	Hylan G-F Single injection	131	60.2 yr (SD ± 11.3)	Kellgren-Lawrence Classification, Grade 1 - 7 (5.3%)<br>Kellgren-Lawrence Classification, Grade 2 - 49 (37.4%)<br>Kellgren-Lawrence Classification, Grade 3 - 68 (51.9%)

Author and Year	Study Design	Interventions	Number of Patients	Mean Age	OA Grade
					Kellgren-Lawrence Classification, Grade 4 - 2 (1.5%)
**Lee S 2004**[42]	Non-Controlled Clinical Trial	Hylan G-F 1-3 weekly injections	74	68.27 yr (SD ± 12.72, Total: 31.9 – 92)	Kellgren-Lawrence Classification, Joint Space, Narrowing, Grade 2 - 67 (91%)<br><br>Kellgren-Lawrence Classification, Osteophytes, Grade 2 - 55 (74%)
**Pal S 2014**[21]	Non-Controlled Clinical Trial	Hylan G-F Single injection	394	57.6 yr (SD ± 9.8)	Osteoarthritis, Knee, Mild to Moderate - 394 (100%)<br><br>Kellgren-Lawrence Classification, Grade 1-3 - 394 (100%)<br><br>Kellgren-Lawrence Classification, Tibiofemoral, Index Knee, Grade 1 - 20 (5.1%)<br><br>Kellgren-Lawrence Classification, Tibiofemoral, Index Knee, Grade 2 - 151 (38.3%)<br><br>Kellgren-Lawrence Classification, Tibiofemoral, Index Knee, Grade 3 - 223 (56.6%)
**Pandey SK 2017**[47]	Observational Non-Comparative	Hylan G-F Single injection	352	59.1 yr (SD ± 4.39)	Kellgren-Lawrence Classification, Grade 2-4 - 352 (100%)
**Raman R 2008**[36]	RCT	Hylan G-F 1-3 weekly injections	199	NR	Kellgren-Lawrence Classification, Grade 3 - 118 (61%)
		Sodium Hyaluronate (Hyalgan®)	193		Kellgren-Lawrence Classification, Grade 3 - 110 (59%)
**Raynauld JP 2002**[22]	RCT	Hylan G-F 1-3 weekly injections	127	62.6 yr (SD ± 9.4)	Kellgren-Lawrence Classification, Grade, Not Reported - 0 (0%)<br><br>Kellgren-Lawrence Classification, Grade 0 - 4 (3%)<br><br>Kellgren-Lawrence Classification, Grade 1 - 17 (13%)<br><br>Kellgren-Lawrence Classification, Grade 2 - 32 (25%)<br><br>Kellgren-Lawrence Classification, Grade 3 - 49 (39%)<br><br>Kellgren-Lawrence Classification, Grade 4 - 25 (20%)
		Appropriate Care	128	63.5 yr (SD ± 10.5)	Kellgren-Lawrence Classification, Grade, Not Reported - 1 (0.78%)<br><br>Kellgren-Lawrence Classification, Grade 0 - 4 (3%)<br><br>Kellgren-Lawrence Classification, Grade 1 - 11 (9%)<br><br>Kellgren-Lawrence Classification, Grade 2 - 33 (26%)<br><br>Kellgren-Lawrence Classification, Grade 3 - 37 (29%)<br><br>Kellgren-Lawrence Classification, Grade 4 - 42 (33%)

Author and Year	Study Design	Interventions	Number of Patients	Mean Age	OA Grade
**Raynauld JP 2005**[37]	RCT	Hylan G-F (Single-Course)	78	63.8 yr (SD ± 9.5)	Kellgren-Lawrence Classification, Grade 0 - 3 (3.9%)<br>Kellgren-Lawrence Classification, Grade 1 - 12 (15.4%)<br>Kellgren-Lawrence Classification, Grade 2 - 22 (28.2%)<br>Kellgren-Lawrence Classification, Grade 3 - 26 (33.3%)<br>Kellgren-Lawrence Classification, Grade 4 - 15 (19.2%)
		Hylan G-F (Repeat-Course)	48	60.8 yr (SD ± 9.2)	Kellgren-Lawrence Classification, Grade 0 - 1 (2.1%)<br>Kellgren-Lawrence Classification, Grade 1 - 5 (10.4%)<br>Kellgren-Lawrence Classification, Grade 2 - 9 (18.8%)<br>Kellgren-Lawrence Classification, Grade 3 - 23 (47.9%)<br>Kellgren-Lawrence Classification, Grade 4 - 10 (20.8%)
		Appropriate Care	128	63.5 yr (SD ± 10.5)	Kellgren-Lawrence Classification, Grade 0 - 4 (3.1%)<br>Kellgren-Lawrence Classification, Grade 1 - 11 (8.6%)<br>Kellgren-Lawrence Classification, Grade 2 - 33 (25.8%)<br>Kellgren-Lawrence Classification, Grade 3 - 37 (28.9%)<br>Kellgren-Lawrence Classification, Grade 4 - 42 (32.8%)
**Rolf CG 2005**[38]	RCT	Hylan G-F 1-3 weekly injections	90	54.5 yr (SD ± 9.16)	Osteoarthritis, Knee, Severe, Symptomatic, Requiring Knee Replacement - 0 (0%)<br>Ahlback Classification, Grade 0 - 36 (40%)<br>Ahlback Classification, Grade 1 - 19 (21%)<br>Ahlback Classification, Grade 2 - 11 (12%)<br>Ahlback Classification, Grade 3 - 2 (2%)
		Hylauron (Artzal®)	91	53.9 yr (SD ± 9)	Osteoarthritis, Knee, Severe, Symptomatic, Requiring Knee Replacement - 0 (0%)<br>Ahlback Classification, Grade 0 - 41 (46%)<br>Ahlback Classification, Grade 1 - 20 (22%)<br>Ahlback Classification, Grade 2 - 11 (12%)

Author and Year	Study Design	Interventions	Number of Patients	Mean Age	OA Grade
					Ahlback Classification, Grade 3 - 1 (1%)
		Placebo	91	53.1 yr (SD ± 10.08)	Osteoarthritis, Knee, Severe, Symptomatic, Requiring Knee Replacement - 0 (0%)<br><br>Ahlback Classification, Grade 0 - 41 (47%)<br><br>Ahlback Classification, Grade 1 - 18 (20%)<br><br>Ahlback Classification, Grade 2 - 7 (8%)<br><br>Ahlback Classification, Grade 3 - 0 (0%)
**Trueba Vasavilbaso C 2017[39]**	RCT	Hylan G-F 1-3 weekly injections	10	56.9 yr (SD ± 9.8)	Kellgren-Lawrence Classification, Grade 1-2 - 10 (100%)<br><br>Kellgren-Lawrence Classification, Grade 1 - 8 (80%)<br><br>Kellgren-Lawrence Classification, Grade 2 - 2 (20%)
		Hylauronic Acid (Orthovisc®) 4 injections	10	71.1 yr (SD ± 7)	Kellgren-Lawrence Classification, Grade 1-2 - 10 (100%)<br><br>Kellgren-Lawrence Classification, Grade 2 - 10 (100%)
		Hylauronic Acid (Suprahyal®/Adant®) 5 injections	10	66.3 yr (SD ± 9.6)	Kellgren-Lawrence Classification, Grade 1-2 - 10 (100%)<br><br>Kellgren-Lawrence Classification, Grade 2 - 6 (60%)
		Platelet Rich Plasma	10	60.3 yr (SD ± 9.5)	Kellgren-Lawrence Classification, Grade 1-2 - 10 (100%)<br><br>Kellgren-Lawrence Classification, Grade 2 - 6 (60%)
		Standard Care	10	67.5 yr (SD ± 7.8)	Kellgren-Lawrence Classification, Grade 1-2 - 10 (100%)<br><br>Kellgren-Lawrence Classification, Grade 2 - 9 (90%)
**Vad VB 2003[43]**	Non-Randomized Controlled Clinical Trial	Hylan G-F 1-3 weekly injections	37	64.8 yr (Total: 41 – 76)	Osteoarthritis, Patellofemoral, Moderate to Severe - 9 (24.32%)<br><br>Kellgren-Lawrence Classification, Grade 1 - 3 (8.11%)<br><br>Kellgren-Lawrence Classification, Grade 2- 6 (16.22%)<br><br>Kellgren-Lawrence Classification, Grade 3 - 17 (45.95%)<br><br>Kellgren-Lawrence Classification, Grade 4 - 11 (29.73%)
		Knee Lavage + Hylan G-F 1-3 weekly injections	41	63.9 yr (Total: 39 – 68)	Osteoarthritis, Patellofemoral, Moderate to Severe - 10 (22.73%)<br><br>Kellgren-Lawrence Classification, Grade 1 - 5 (11.36%)<br><br>Kellgren-Lawrence Classification, Grade 2 - 6 (13.64%)<br><br>Kellgren-Lawrence Classification, Grade 3 - 21 (47.73%)

Author and Year	Study Design	Interventions	Number of Patients	Mean Age	OA Grade
					Kellgren-Lawrence Classification, Grade 4 - 12 (27.27%)
**Waddell DD 2005**[44]	Non-Controlled Clinical Trial	Hylan G-F 1-3 weekly injections	71	65.5 yr (SD ± 11.1, Total: 40 – 87)	Kellgren-Lawrence Classification, Grade 2 - 1 (1.4%)<br>Kellgren-Lawrence Classification, Grade 3 - 18 (25.4%)<br>Kellgren-Lawrence Classification, Grade 4 - 52 (73.2%)
**Yan CH 2015**[48]	Observational Non-Comparative	Hylan G-F Single injection	95	62 yr (SD ± 9.8, Total: 33 – 86)	Kellgren-Lawrence Classification, Grade 1 - 5 (5.26%)<br>Kellgren-Lawrence Classification, Grade 2 - 30 (31.58%)<br>Kellgren-Lawrence Classification, Grade 3 - 38 (40%)<br>Kellgren-Lawrence Classification, Grade 4 - 37 (38.95%)

NR= not reported, yr= year

Table S5 Efficacy outcomes

Author and Year	Interventions	WOMAC				VAS Pain	SF-36 MCS	SF-36 PCS
		Total	Pain	Physical Function	Stiffness			
**Acharya KKV 2013**[27]	Hylan G-F Single injection	WOMAC - 32 units	--	--	--	VAS, Pain, % Change - -87%	--	--
	Arthroscopy	WOMAC - 62 units	--	--	--	VAS, Pain, % Change - -42%	--	--
**Atamaz F 2006**[28]	Hylan G-F 1-3 weekly injections	--	WOMAC, Pain, Likert - 10.2 units (SD ± 2.9)	WOMAC, Physical Function, Likert - 38.9 units (SD ± 11.7)	--	VAS, Pain, Spontaneous - 49 mm (SD ± 13.3)	--	SF-36, Physical Functioning - 54 units (SD ± 25.9)
	Sodium hyaluronate + sodium chloride (Orthovisc®)	--	WOMAC, Pain, Likert - 11.2 units (SD ± 3.5)	WOMAC, Physical Function, Likert - 37.6 units (SD ± 13.6)	--	VAS, Pain, Spontaneous - 56.7 mm (SD ± 23)	--	SF-36, Physical Functioning - 31 units (SD ± 14.3)
	Physical Therapy Agents	--	WOMAC, Pain, Likert - 15.5 units (SD ± 4.1)	WOMAC, Physical Function, Likert - 38.2 units (SD ± 12.6)	--	VAS, Pain, Spontaneous - 59.5 mm (SD ± 21.2)	--	SF-36, Physical Functioning - 53 units (SD ± 22.7)
**Clarke S 2005**[40]	Hylan G-F 1-3 weekly injections	--	WOMAC, Pain, VAS - 205 mm (SD ± 133.24, 95% CI: 150 – 260)<br><br>WOMAC, Pain, VAS, % Change - -26%<br><br>WOMAC, Pain, Stair Climbing, VAS - 50.7 mm (SD ± 30.28, 95% CI: 38.2 – 63.2)	WOMAC, Physical Function, VAS - 700 mm (SD ± 462.69, 95% CI: 509 – 891)	WOMAC, Stiffness, VAS - 89 mm (SD ± 60.56, 95% CI: 64 – 114)	--	--	--
**Cole BJ 2017**[31]	Hylan G-F 1-3 weekly injections	--	WOMAC, Pain - 4 units (SD ± 4.24, SE ± 0.6)	--	--	VAS, Pain - 57.3 mm (SD ± 26.87, SE ± 3.8)	--	--
	Platelet-Rich Plasma	--	WOMAC, Pain - 3.02 units (SD ± 3.36, SE ± 0.48)	--	--	VAS, Pain - 44 mm (SD ± 32.2, SE ± 4.6)	--	--

Author and Year	Interventions	WOMAC				VAS Pain	SF-36 MCS	SF-36 PCS
		Total	Pain	Physical Function	Stiffness			
**Daniel D 2011**[45]	Hylan G-F Single injection	--	--	--	--	--	--	--
**Huskin JP 2008**[41]	Hylan G-F 1-3 weekly injections	WOMAC - 20.6 units	WOMAC, Pain - 3.8 units	WOMAC, Physical Function - 14.2 units	WOMAC, Stiffness - 1.4 units	VAS, Pain, Walking - 26.8 mm	--	--
**Karlsson J 2002**[34]	Hylan G-F 1-3 weekly injections	--	--	--	--	VAS, Pain, Maximum, Change - -22.3 mm	--	--
	1% hyaluronan (Artzal®) – 3 injections per week	--	--	--	--	VAS, Pain, Maximum, Change - -23.5 mm	--	--
	Placebo	--	--	--	--	VAS, Pain, Maximum, Change - Baseline – -21.9 mm	--	--
**Kearey P 2016**[46]	Hylan G-F Single injection	WOMAC, Change - -18.19 units (SD ± 23.6)<br><br>WOMAC, % Change - -33.94%	WOMAC, Pain, % Change - -36.77%<br><br>WOMAC, Pain, Change - -19.78 units (SD ± 24.28)	WOMAC, Physical Function, Change - 17.55 units (SD ± 23.9)<br><br>WOMAC, Physical Function, % Change - 33.12%	WOMAC, Stiffness, Change - -20.25 units (SD ± 26.18)<br><br>WOMAC, Stiffness, % Change - -34.77%	--	SF-36, Mental Component - 45.79 units (SD ± 13.84)<br><br>SF-36, Mental Component, Change - 1.48 units (SD ± 11.58)<br><br>SF-36, Mental Component, % Change - 3.34%	SF-36, Physical Component - 35.47 units (SD ± 10.29)<br><br>SF-36, Physical Component, Change - 3 units (SD ± 10.17)<br><br>SF-36, Physical Component, % Change - 9.24%
**Pal S 2014**[21]	Hylan G-F Single injection	--	WOMAC, Pain, VAS - 27 mm (SD ± 17.1)<br><br>WOMAC, Pain, VAS, Change - -29.2 mm (SD ± 19.2)<br><br>WOMAC, Pain, Walking, VAS - 27.8	WOMAC, Physical Function, VAS - 28.7 mm (SD ± 17.8)<br><br>WOMAC, Physical Function, VAS, Change - -25.7 mm (SD ± 19.5)	WOMAC, Stiffness, VAS - 27 mm (SD ± 18.6)<br><br>WOMAC, Stiffness, VAS, Change - -25.7 mm (SD ± 22)	--	--	--

Author and Year	Interventions	WOMAC				VAS Pain	SF-36 MCS	SF-36 PCS
		Total	Pain	Physical Function	Stiffness			
			mm (SD ± 18.1)<br><br>WOMAC, Pain, Walking, VAS, Change - -32.7 mm (SD ± 20)<br><br>WOMAC, Pain, Walking, VAS, % Change - -54%					
**Pandey SK 2017**[47]	Hylan G-F Single injection	WOMAC, Likert - 36 units (SD ± 11.65)	--	--	--	--	--	--
**Raman R 2008**[36]	Hylan G-F 1-3 weekly injections	--	WOMAC, Pain, Likert - 5.8 units	WOMAC, Physical Function, Likert - 15 units	--	VAS, Pain - 3.5 cm	--	--
	Sodium Hyaluronate (Hyalgan®)	--	WOMAC, Pain, Likert - 8.5 units	WOMAC, Physical Function, Likert - 33.3 units	--	VAS, Pain - 6.4 cm	--	--
**Raynauld JP 2002**[22]	Hylan G-F 1-3 weekly injections	--	WOMAC, Pain, Likert - 6.94 units (SD ± 3.97)<br><br>WOMAC, Pain, Likert, Change - -4.41 units (SD ± 3.88)<br><br>WOMAC, Pain, Likert, % Change - -38.41% (SD ± 34.39)	WOMAC, Physical Function, Likert - 24.26 units (SD ± 12.95)<br><br>WOMAC, Physical Function, Likert, Change - -15.04 units (SD ± 12.29)<br><br>WOMAC, Physical Function, Likert, % Change - -37.82% (SD ± 31.44)	WOMAC, Stiffness - 3.22 units (SD ± 1.74)<br><br>WOMAC, Stiffness, Change - -1.83 units (SD ± 1.73)<br><br>WOMAC, Stiffness, % Change - -34.74% (SD ± 35)	--	SF-36, Mental Component - 55.29 units (SD ± 10.45)<br><br>SF-36, Mental Component, Change - 3.32 units (SD ± 12.06)<br><br>SF-36, Mental Component, % Change - 11.53% (SD ± 33.8)	SF-36, Physical Component 33.24 units (SD ± 10.16)<br><br>SF-36, Physical Component, Change - 4.88 units (SD ± 9.78)<br><br>SF-36, Physical Component, % Change - 20.31% (SD ± 37.43)
	Appropriate Care	--	WOMAC, Pain, Likert - 10.1 units (SD ± 4.24) - (N = 107)<br><br>WOMAC, Pain, Likert,	WOMAC, Physical Function, Likert - 33.87 units (SD ± 13.88)<br><br>WOMAC, Physical Function, Likert,	WOMAC, Stiffness - 4.31 units (SD ± 1.56)<br><br>WOMAC, Stiffness, Change - -0.71 units (SD ± 1.57) - (N = 107)	--	SF-36, Mental Component - 52.65 units (SD ± 11.56)<br><br>SF-36, Mental Component, Change - 1.55	SF-36, Physical Component - 27.78 units (SD ± 8.9)<br><br>SF-36, Physical Component,

Author and Year	Interventions	WOMAC				VAS Pain	SF-36 MCS	SF-36 PCS
		Total	Pain	Physical Function	Stiffness			
			Change - -1.83 units (SD ± 3.83)<br><br>WOMAC, Pain, Likert, % Change - -13.34% (SD ± 39.86)	Change - -5.85 units (SD ± 11.18)<br><br>WOMAC, Physical Function, Likert, % Change - -14.52% (SD ± 30.39)	WOMAC, Stiffness, % Change - -10.42% (SD ± 37.42) - (N = 107)		units (SD ± 10.55)<br><br>SF-36, Mental Component, % Change - 5.4% (SD ± 23.11)	Change - -0.4 units (SD ± 7.22)<br><br>SF-36, Physical Component, % Change - 1.07% (SD ± 29.1)
**Raynauld JP 2005**[37]	Hylan G-F (Single-Course)	--	WOMAC, Pain - 6.71 units	WOMAC, Physical Function, % Change - -39.6%	WOMAC, Stiffness, % Change - -35.8%	--	SF-36, Mental Component, % Change - 9.8%	SF-36, Physical Component, % Change - 19.7%
	Hylan G-F (Repeat-Course)	--	WOMAC, Pain - 7.44 units	WOMAC, Physical Function, % Change - -35%	WOMAC, Stiffness, % Change - -33%	--	SF-36, Mental Component, % Change - 14.3%	SF-36, Physical Component, % Change - 21.3%
	Appropriate Care	--	WOMAC, Pain - 10.1 units	WOMAC, Physical Function, % Change - -14.5%	WOMAC, Stiffness, % Change - -10.4%	--	SF-36, Mental Component, % Change - 5.4%	SF-36, Physical Component, % Change - 1.1%
**Trueba Vasavilbaso C 2017**[39]	Hylan G-F 1-3 weekly injections	WOMAC, Likert - 18.6 units (SD ± 8.55)<br><br>WOMAC, Likert, % Change - -67.12% (± 12.82)	WOMAC, Pain, Likert, % Change - -48.48%	WOMAC, Physical Function, Likert, % Change - -72.82%	WOMAC, Stiffness, Likert, % Change - -50%	--	--	--
	Hylauronic Acid (Orthovisc®) 4 injections	WOMAC, Likert - 51.4 units (SD ± 27.46)<br><br>WOMAC, Likert, % Change - -21.44% (± 45.94)	WOMAC, Pain, Likert, % Change - -21.71%	WOMAC, Physical Function, Likert, % Change - -23.59%	WOMAC, Stiffness, Likert, % Change - 39%	--	--	--
	Hylauronic Acid (Suprahyal®/Adant®) 5 injections	WOMAC, Likert - 17.2 units (SD ± 13.07)<br><br>WOMAC, Likert, % Change - -71.79% (± 14.91)	WOMAC, Pain, Likert, % Change - -71.07%	WOMAC, Physical Function, Likert, % Change - -71.06%	WOMAC, Stiffness, Likert, % Change - -80%	--	--	--
	Platelet Rich Plasma	WOMAC, Likert - 30 units (SD ± 35.11)<br><br>WOMAC, Likert, % Change - -48.84% (± 58.11)	WOMAC, Pain, Likert, % Change - -32.42%	WOMAC, Physical Function, Likert, % Change – -51.13%	WOMAC, Stiffness, Likert, % Change - -35%	--	--	--

Author and Year	Interventions	WOMAC				VAS Pain	SF-36 MCS	SF-36 PCS
		Total	Pain	Physical Function	Stiffness			
	Standard Care	WOMAC, Likert – 53.5 units (SD ± 16.5)<br><br>WOMAC, Likert, % Change - -10.28% (± 50.08)	WOMAC, Pain, Likert, % Change - 29.44%	WOMAC, Physical Function, Likert, % Change - -41.46%	WOMAC, Stiffness, Likert, % Change - 55%	--	--	--
**Vad VB 2003**[43]	Hylan G-F 1-3 weekly injections	--	--	--	--	VAS, Pain - 3.2 cm (± 1.2)	--	--
	Knee Lavage + Hylan G-F 1-3 weekly injections	--	--	--	--	VAS, Pain - 2.1 cm (± 1.4)	--	--
**Waddell DD 2005**[44]	Hylan G-F 1-3 weekly injections	WOMAC, Change - -19.8 units (SD ± 15.21, SE ± 2.13)	WOMAC, Pain, Walking - 1.25 units (SD ± 0.79, SE ± 0.11)<br><br>WOMAC, Pain, Walking, Change - -1.1 units (SD ± 0.86, SE ± 0.12)	WOMAC, Physical Function, Change - -13.69 units (SD ± 11, SE ± 1.54)	--	--	--	--
**Yan CH 2015**[48]	Hylan G-F Single injection	--	--	--	--	VAS, Pain - 48 mm (SD ± 20.6, Total: 0 – 85)	--	--

Table S6 Safety outcomes

Author and Year	Interventions	Adverse Events				Drug Discontinuation due to AE	Study Withdrawal due to AE
		Total	Serious	Treatment-Related	Target Knee		
**Atamaz F 2006[28]**	Hylan G-F 1-3 weekly injections	--	--	Adverse Events, Treatment-Related, Severe - 0 (0%)	--	--	--
	Sodium hyaluronate + sodium chloride (Orthovisc®)	--	--	Adverse Events, Treatment-Related, Severe - 0 (0%)	--	--	--
	Physical Therapy Agents	--	--	--	--	--	--
**Boutefnouchet T 2017[49]**	Hylan G-F Single injection	--	--	Adverse Events, Treatment-Related - 0 (0%)	--	--	--
**Clarke S 2005[40]**	Hylan G-F 1-3 weekly injections	--	--	--	--	Drug Discontinuation, Adverse Events - 2 (4.65%)	--
**Daniel D 2011[45]**	Hylan G-F Single injection	Adverse Events - 15 (2.63%)	Adverse Events, Serious - 0 (0%)	Adverse Events, Treatment-Related, Local - 11 (1.93%)	--	--	--
**Huskin JP 2008[41]**	Hylan G-F 1-3 weekly injections	--	--	Adverse Events, Treatment-Related, Serious, Target Knee - 1 (1.6%)<br><br>Adverse Events, Treatment-Related, Target Knee - 12 (19%)	--	--	Study Withdrawal, Adverse Events - 6 (9.68%)<br><br>Study Withdrawal, Adverse Events, Treatment-Related, Possible, Target Knee - 3 (4.84%)
**Karatosun V 2005[33]**	Hylan G-F 1-3 weekly injections	--	--	Adverse Events, Treatment-Related, Major - 0 (0%)<br><br>Adverse Events, Treatment-Related - 0 (0%)	--	--	--
	Low-Molecular Weight Hyaluronic acid - 3 injections (Orthovisc®)	--	--	Adverse Events, Treatment-Related, Major - 0 (0%)	--	--	--

Author and Year	Interventions	Adverse Events				Drug Discontinuation due to AE	Study Withdrawal due to AE
		Total	Serious	Treatment-Related	Target Knee		
				Adverse Events, Treatment-Related - 0 (0%)			
**Karlsson J 2002**[34]	Hylan G-F 1-3 weekly injections	--	--	Adverse Events, Treatment-Related, Serious - 0 (0%)	--	Drug Discontinuation, Adverse Events - 1 (1.16%)	--
	1% hyaluronan (Artzal®) – 3 injections per week	--	--	Adverse Events, Treatment-Related, Serious - 0 (0%)	--	Drug Discontinuation, Adverse Events - 2 (2.22%)	--
	Placebo	--	--	Adverse Events, Treatment-Related, Serious - 0 (0%)	--	Drug Discontinuation, Adverse Events - 2 (3.03%)	--
**Kearey P 2016**[46]	Hylan G-F Single injection	Adverse Events - 37 (28%)	--	--	--	--	--
**Lee S 2004**[42]	Hylan G-F 1-3 weekly injections	--	--	Adverse Events, Treatment-Related, Localized - 3 (4%)	--	--	--
**Pal S 2014**[21]	Hylan G-F Single injection	--	Adverse Events, Serious - 6 (2%)	Adverse Events, Treatment-Related - 10 (3%)	Adverse Events, Target Knee - 23 (5.8%)	--	Study Withdrawal, Adverse Events - Baseline – 52 1 (0.25%)
**Raman R 2008**[36]	Hylan G-F 1-3 weekly injections	--	Adverse Events, Serious - 1 (0.52%)	Adverse Events, Treatment-Related - 39 (20.1%)	--	--	Study Withdrawal, Adverse Events - 0 (0%)
	Sodium Hyaluronate (Hyalgan®)	--	Adverse Events, Serious - 1 (0.52%)	Adverse Events, Treatment-Related - 30 (16.13%)	--	--	Study Withdrawal, Adverse Events - 0 (0%)
**Raynauld JP 2002**[22]	Hylan G-F 1-3 weekly injections	Adverse Events - 122 (96%)	Adverse Events, Serious - 1 (0.52%)	--	--	--	--
	Appropriate Care	Adverse Events - 115 (90%)	Adverse Events, Serious - 1 (0.52%)	--	--	--	--
**Raynauld JP 2005**[37]	Hylan G-F (Single-Course)	--	--	Adverse Events, Treatment-Related, Target Knee - 1 (1.28%)<br><br>Adverse Events, Treatment-Related, First Course, Target Knee - 1 (1.28%)	Adverse Events, First Course, Target Knee - 19 (24%)	--	--

Author and Year	Interventions	Adverse Events				Drug Discontinuation due to AE	Study Withdrawal due to AE
		Total	Serious	Treatment-Related	Target Knee		
	Hylan G-F (Repeat-Course)	--	--	Adverse Events, Treatment-Related, Target Knee - 5 (10.42%)<br><br>Adverse Events, Treatment-Related, First Course, Target Knee - 2 (4.17%)<br><br>Adverse Events, Treatment-Related, Second Course, Target Knee - 3 (6.25%)	Adverse Events, First Course, Target Knee - 11 (22.92%)<br><br>Adverse Events, Second Course, Target Knee - 13 (27.08%)	--	--
	Appropriate Care	--	--	--	--	--	--
**Rolf CG 2005**[38]	Hylan G-F 1-3 weekly injections	Adverse Events - 53 (59%)	--	Adverse Events, Treatment-Related, Severe - 1 (1.11%)<br><br>Adverse Events, Treatment-Related, Serious - 0 (0%)	--	--	--
	Hylauron (Artzal®)	Adverse Events - 55 (60%)	--	Adverse Events, Treatment-Related, Severe - 4 (4.4%)<br><br>Adverse Events, Treatment-Related, Serious - 0 (0%)	--	--	--
	Placebo	Adverse Events - 55 (60%)	--	Adverse Events, Treatment-Related, Severe - 1 (1.1%)<br><br>Adverse Events, Treatment-Related, Serious - 0 (0%)	--	--	--
**Trueba Vasavilbaso C 2017**[39]	Hylan G-F 1-3 weekly injections	--	--	Adverse Events, Treatment-Related - 0 (0%)	--	--	--
	Hylauronic Acid (Orthovisc®) 4 injections	--	--	Adverse Events, Treatment-Related - 0 (0%)	--	--	--

Author and Year	Interventions	Adverse Events				Drug Discontinuation due to AE	Study Withdrawal due to AE
		Total	Serious	Treatment-Related	Target Knee		
	Hylauronic Acid (Suprahyal®/Adant®) 5 injections	--	--	Adverse Events, Treatment-Related - 0 (0%)	--	--	--
	Platelet Rich Plasma	--	--	Adverse Events, Treatment-Related - 0 (0%)	--	--	--
	Standard Care	--	--	Adverse Events, Treatment-Related - 0 (0%)	--	--	--
**Waddell DD 2005**[44]	Hylan G-F 1-3 weekly injections		--	Adverse Events, Treatment-Related, Possible - 3 (4.2%)<br><br>Adverse Events, Treatment-Related, Probable - 3 (4.2%)<br><br>Adverse Events, Treatment-Related, Definite - 7 (9.9%)	--	--	Study Withdrawal, Adverse Events, Treatment-Related - 0 (0%)
**Yan CH 2015**[48]	Hylan G-F Single injection	Adverse Events - 18 (16.4%)	--	--	--	--	--

Table S7 Cochrane Collaboration tool

Domain	Support for judgment	Review authors' judgement
**Selection bias**		
Random sequence generation.	Describe the method used to generate the allocation sequence in sufficient detail to allow an assessment of whether it should produce comparable groups.	Selection bias (biased allocation to interventions) due to inadequate generation of a randomized sequence.
Allocation concealment.	Describe the method used to conceal the allocation sequence in sufficient detail to determine whether intervention allocations could have been foreseen in advance of, or during, enrolment.	Selection bias (biased allocation to interventions) due to inadequate concealment of allocations prior to assignment.
**Performance bias**		
Blinding of participants and personnel<br>Assessments should be made for each main outcome (or class of outcomes).	Describe all measures used, if any, to blind study participants and personnel from knowledge of which intervention a participant received. Provide any information relating to whether the intended blinding was effective.	Performance bias due to knowledge of the allocated interventions by participants and personnel during the study.
**Detection bias**		
Blinding of outcome assessment<br>Assessments should be made for each main outcome (or class of outcomes).	Describe all measures used, if any, to blind outcome assessors from knowledge of which intervention a participant received. Provide any information relating to whether the intended blinding was effective.	Detection bias due to knowledge of the allocated interventions by outcome assessors.
**Attrition bias**		
Incomplete outcome data<br>Assessments should be made for each main outcome (or class of outcomes).	Describe the completeness of outcome data for each main outcome, including attrition and exclusions from the analysis. State whether attrition and exclusions were reported, the numbers in each intervention group (compared with total randomized participants), reasons for attrition/exclusions where reported, and any re-inclusions in analyses performed by the review authors.	Attrition bias due to amount, nature or handling of incomplete outcome data.
**Reporting bias**		
Selective reporting	State how the possibility of selective outcome reporting was examined by the review authors, and what was found.	Reporting bias due to selective outcome reporting.
**Other bias**		
Other sources of bias	State any important concerns about bias not addressed in the other domains in the tool. If particular questions/entries were pre-specified in the review's protocol, responses should be provided for each question/entry.	Bias due to problems not covered elsewhere in the table.

Table S8 Cochrane quality assessment summary

Author & Year	Selection Bias		Performance Bias	Detection Bias	Attrition Bias	Reporting Bias	Other Bias
	Random Sequence Generation	Allocation Concealment	Blinding of Participants and Personnel	Blinding of Outcome Assessment	Incomplete Outcome Data	Selective Outcome Reporting	Other Source of Bias
Acharya KKV 2013[27]	Unclear	Unclear	Unclear	Unclear	Unclear	Unclear	Low
Atamaz F 2006[28]	Unclear	Unclear	High	High	High	Low	High
Cole BJ 2017[31]	Unclear	Unclear	Low	Low	High	Low	Low
Juni P 2007[32]	Low	Low	High	Low	Low	Low	High
Karatosun V 2005[33]	Low	Low	Low	Low	High	Low	Low
Karlsson J 2002[34]	Low	Low	Low	Low	High	Low	High
Raman R 2008[36]	Low	Low	High	Low	High	Low	Low
Raynauld JP 2002[22]	Low	Low	High	High	High	Low	High
Rolf CG 2005[38]	Unclear	Unclear	Low	Low	High	Low	High
Trueba Vasavilbaso C 2017[39]	Low	Unclear	High	Low	High	Low	Low

Table S9 Newcastle-Ottawa Scale

**Study Design**	**Selection**				**Comparability**	**Outcome/Exposure**		
**Cohort**	Representativeness of the exposed cohort	Selection of the non-exposed cohort	Ascertainment of exposure	Demonstration that outcome of interest was not present at start of study	Comparability of cohorts on the basis of the design or analysis	Assessment of outcome	Was follow-up long enough for outcomes to occur	Adequacy of follow-up of cohorts
**Case Control**	Is the case definition adequate	Representativeness of the cases	Selection of controls	Definition of controls	Comparability of cases and controls on the basis of the design or analysis	Ascertainment of exposure	Same method of ascertainment of cases and controls	Non-response rate

Table S10 Newcastle-Ottawa Scale quality assessment

Author & Year	Selection	Comparability	Exposure or Outcome	Final Score
Clarke S 2005[40]	**		*	3
Daniel D 2011[45]	**		*	3
Huskin JP 2008[41]	**		*	3
Kearey P 2016[46]	**	**	*	5
Lee S 2004[42]	***		*	4
Pal S 2014[21]	***	**	**	7
Pandey SK 2017[47]	*	*	*	3
Vad VB 2003[43]	***	*	**	6
Waddell DD 2005[44]	**	**	*	5
Yan CH 2015[48]	**		**	4

The Newcastle-Ottawa Scale is an instrument that is used to evaluate three domains of bias: selection of study groups, comparability of the groups, and assessment of outcome/exposure (**Table S9**).[25] Each high-quality item was allocated with a star. A maximum of one star can be designated for each item within the "Selection" and "Outcome/Exposure" and a maximum of two stars for "Comparability." The sum of stars for each study can range from 0 to 9 stars. Studies are rated as low (0-3 stars), moderate (4-6 stars), or high (7-9 stars) quality.

The quality of 10 non-RCT studies were assessed with the Newcastle-Ottawa Scale (**Table S10**). Boutefnouchet et al. 2017[5] is a case series and was not assessed using the Newcastle-Ottawa Scale as the scale is designed for cohort and case control studies. Overall, studies were mainly rated as moderate quality. Nine studies (90%) were rated as moderate quality, with 6 studies (60%) receiving a final score of 4,[6,8,9,14,16,24] 1 study (10%) receiving a final score of 5,[23] and 2 studies (20%) receiving a final score of 6.[13,22] One study (10%) was rated as high quality with a final score of 7.[15] For the Selection criteria, the majority of studies (7 studies, 70%) were allocated 3 stars. [6,8,9,13-15,22] Three studies (30%) were allocated 2 stars.[16,23,24] For the Comparability criteria, the majority of the studies (5 studies, 50%) were allocated 0 stars.[6,8,9,14,24]

Two studies (20%) were allocated 1 star[16] and three studies (30%) were allocated 2 stars.[15,23] For the Exposure/Outcome criteria, the majority of the studies (7 studies, 70%) were allocated 1 star[6,8,9,13,14,16,23] and the remaining three studies were allocated 2 stars.[15,22,24]

Table S11 List of Adverse Events Reported Across Included Studies

Type of Adverse Event	Studies Reporting AE
Adverse Events, Overall	2,5,6,8,12,13,18,20,24
Adverse Events, Treatment Related	11,12,13,17,21
Anaphylactic shock	10
Arthralgia	8,15,20,23
Arthritis	23
Arthropathy	20
Arthrosis	23
Asthmatic Crisis	8
Back Pain	20
Bacterial Infection	20
Burning Sensation	8
Bursitis	15
Cerebrovascular Accident	6
Chills	8
CNS Disorders	8,10,20
Coryzal Symptoms	6
Effusion	10
Endocrine and Metabolic Disorders	10
Flare	10
Flushing	8
Foot Pain	6
General Disorders and Administration Site Conditions	8,10
GI	8,10,18,20
GI, Severe	18
GI, Severe, Treatment Related	18
Headache	20
Hypersensitivity	8
Hyperventilation	8
Hypotension	8
Immune System Disorders	8
Infected toenail	6
Infection	20
Influenza-like Symptoms	20

Type of Adverse Event	Studies Reporting AE
Injection Site Reaction (total)	2,6,14,15,20
Injection Site Reaction, Mild	6
Injection Site Reaction, Moderate or Severe	6
Joint Effusion	8
Joint or Musuloskeletal Stiffness	6,8,15
Joint Swelling	8,24
Local AE, Requiring Additional Treatment	10
Local or Taget Knee AE, Moderate to Severe	15
Local or Target Knee AE	10,13,15,18,19,22
Local or Target Knee AE, Treatment Related	8,9,13,15,18,19,23
Mental and Behavioral Disorders	10
Mild Adverse Events	8,17,24
Mild Adverse Events, Treatment Related	13
Moderate Adverse Events	8
Moderate Adverse Events, Treatment Related	13
MSK Disorders	8,10,20
Myalgia	8
Nausea	6,8
Neck pain	6
Neoplasms	10
Pain	8,20,24
Pain in Contralateral Knee	6
Polycythaemia rubra vera	6
Respiratory System Disorder	8,10,20
Rheumatoid Arthritis	6
Rhinitis	20
Septic Arthritis	10
Severe/Serious Adverse Events	2,8,10,12,13,15,17,18,24
Severe/Serious Adverse Events, Treatment-Related	10,13,20
Sinusitis	20
Skeletal Pain	20
Syncope	8
Synovitis	8,9,15
Systemic Adverse Events	2,14,15,17,20

Type of Adverse Event	Studies Reporting AE
Systemic Adverse Events, Treatment Related	[15]
Target Knee AE, Mild or Moderate, Treatment Related	[9,13,19,23]
Target Knee AE, Severe, Treatment Related	[9,13,23]
Toothache	[20]
Urinary System Disorder	[10,20]
UTI	[20]
Vascular Disorders	[8,10]
Vascular Disorders	[8]
Vertigo	[8]
Viral Infection	[20]

Figure S1 PRISMA diagram

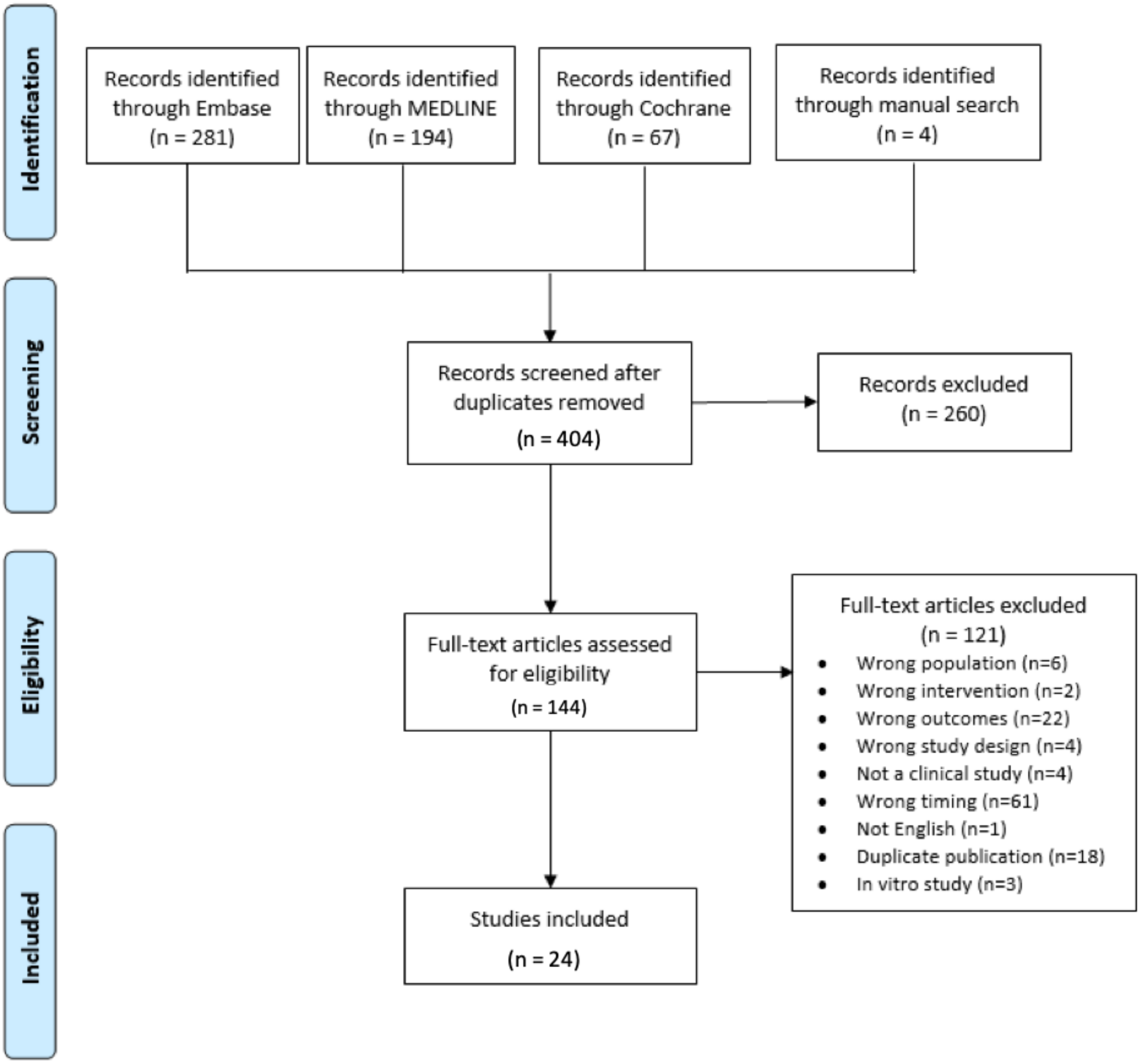

Gap search results from October 18, 2018 to August 17, 2020

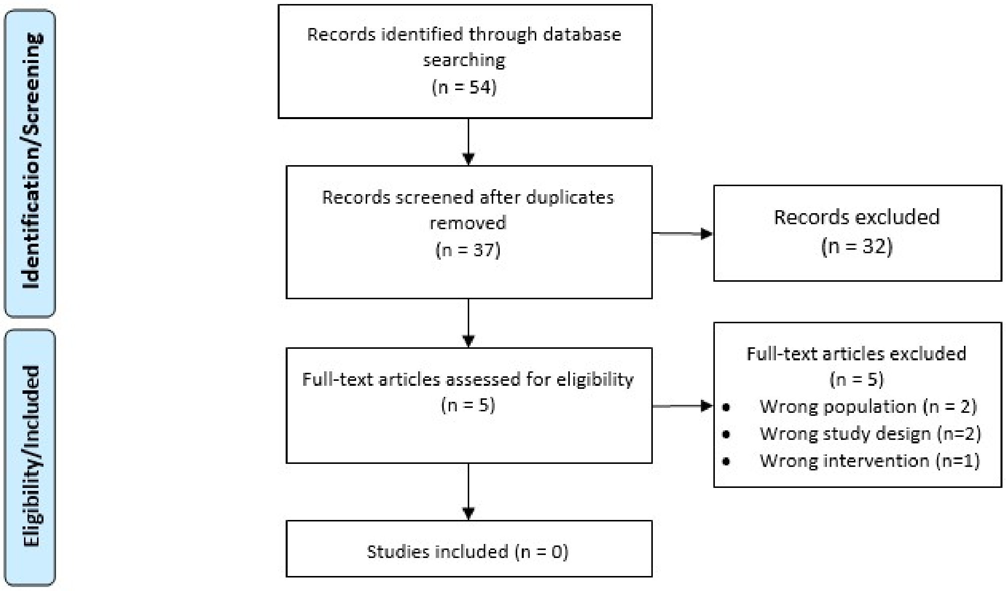

Figure S2 Cochrane quality assessment graph

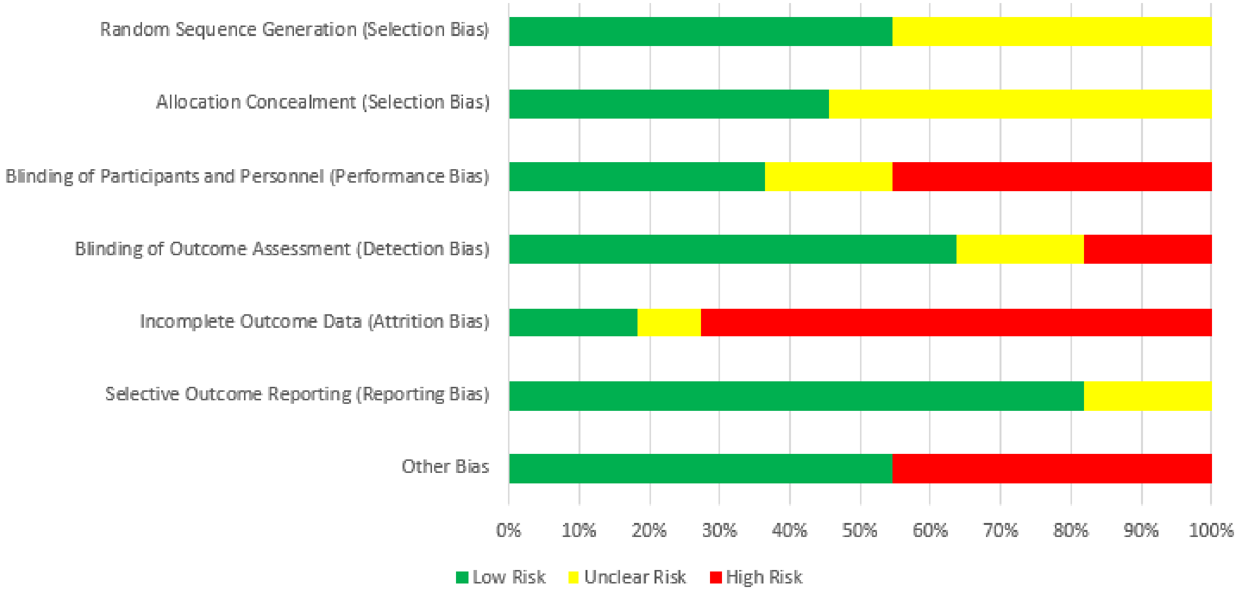

The Cochrane Collaboration's tool is an instrument that is used to evaluate seven domains of bias: random sequence generation (selection bias), allocation concealment (selection bias), blinding of participants and personnel (performance bias), blinding of outcome assessment (detection bias), incomplete outcome data (attrition bias), selective reporting (reporting bias), and other sources of bias (other bias) (**Table S7**).[26]

Risk of bias assessments were conducted on 10 trials (13 publications) (**Table S8** and **Figure S2**). The risk of selection bias was mostly low risk for random sequence generation (60%, 6 trials) and unclear risk for allocation concealment (50%, 5 trials); there were no trials assessed as high risk. For performance bias, pertaining to the blinding of participants and personnel, 50% (5 trials) were assessed as high risk as they were single-blinded or open-label studies, 40% (4 trials) were assessed as low risk, and 20% (2 trials) were assessed as unclear risk. The risk of detection bias, pertaining to the blinding of outcome assessment was mainly low (70%, 7 studies); however, 20% (2 trials) were assessed as high risk as they were single-blinded or open-label studies. For attrition bias, pertaining to incomplete outcome data, 80% (8 trials) were assessed

as high risk as studies had high rates of dropouts, differential rates of dropouts between groups, and/or missing data have not been imputed using appropriate methods; 10% (1 trial) was assessed as low risk and 10% (1 trial) was assessed as unclear risk. The risk of reporting bias, pertaining to selective outcome reporting, was mainly low (90%, 9 studies) and there were no trials assessed as high risk. The risk of other bias was mainly low (50%, 5 trials); however, 50% (5 trials) were assessed as high risk as the studies allowed retreatment and/or concomitant medication, which may have skewed the outcome estimates.

Figure S3 WOMAC Stiffness (non-RCTs)

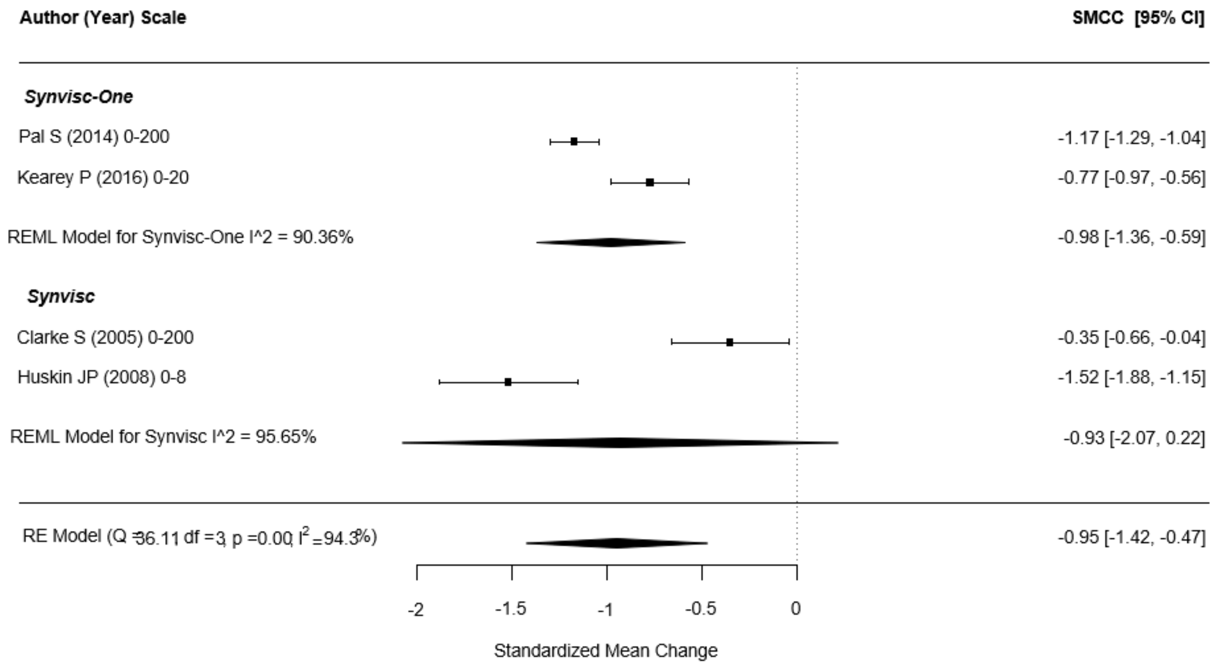

Figure S4 WOMAC Stiffness (RCTs)

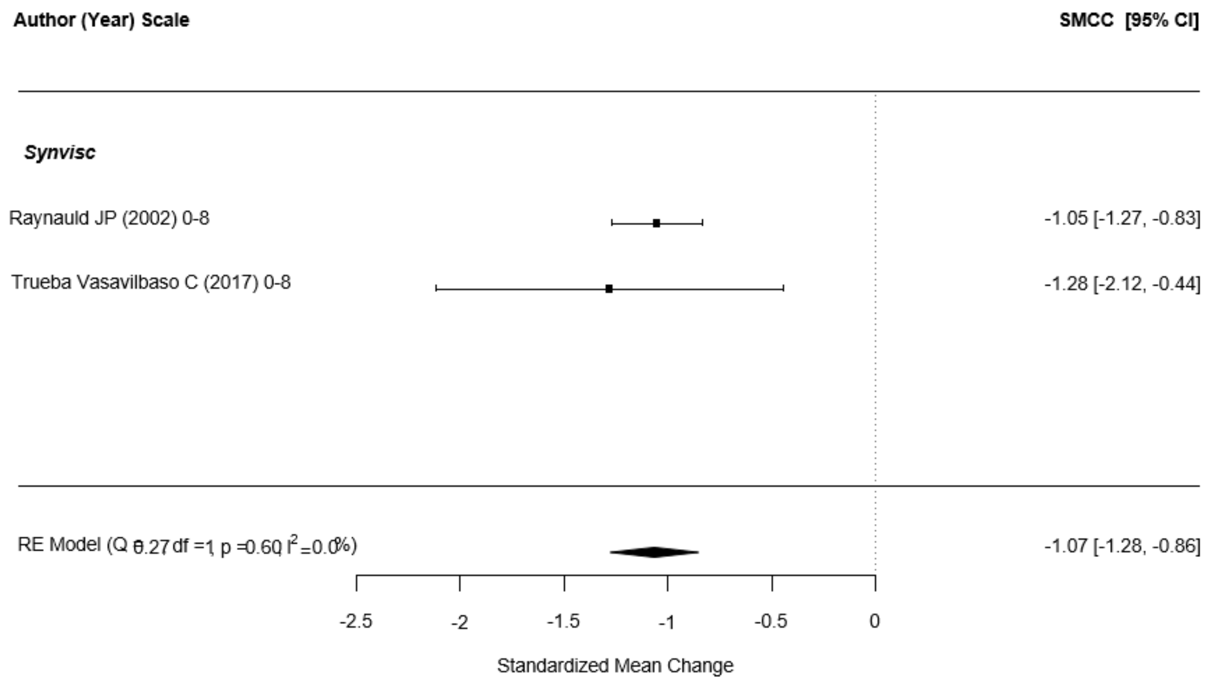

Figure S5 VAS Pain (non-RCTs)

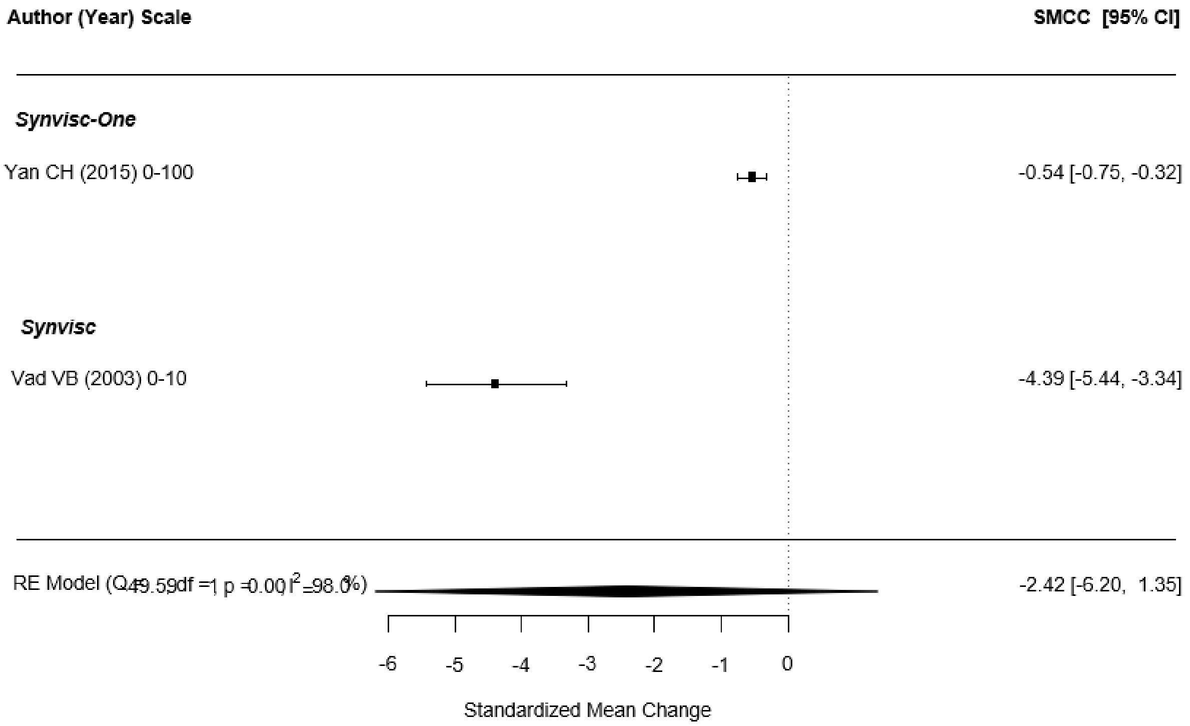

Figure S6 VAS Pain (RCTs)

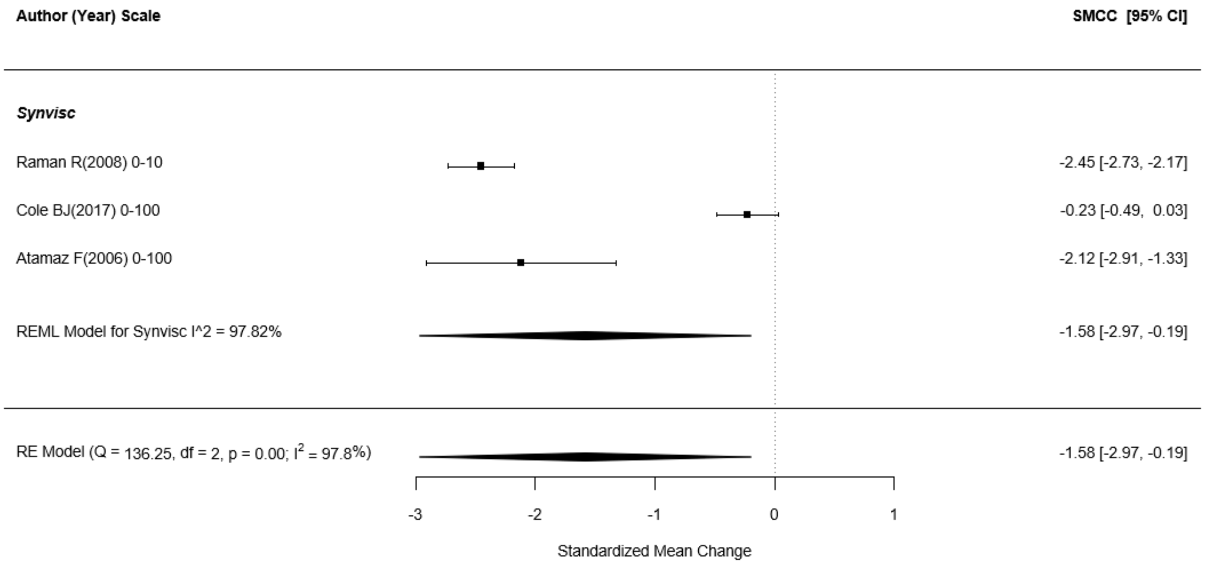

Figure S7 Adverse Events (Non-RCTs)

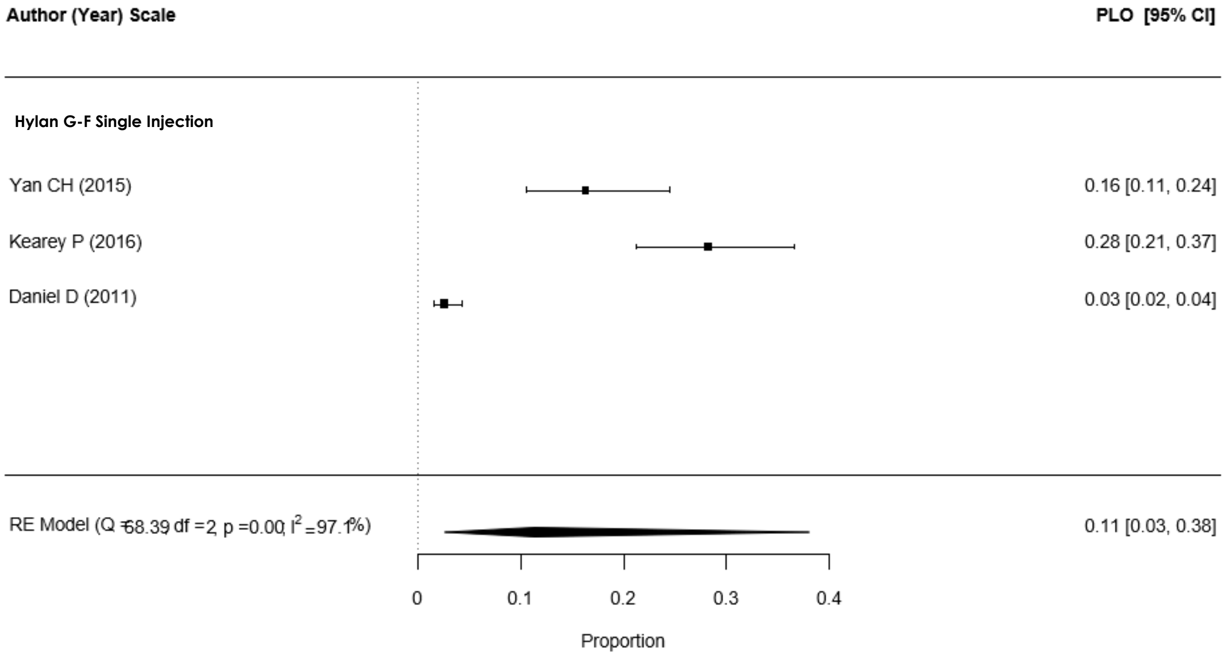

Across 3 non-RCT studies with 812 participants, 11% (95% CI 3, 38; p=0.0101) of those that used single Hylan G-F injection experienced an adverse event.[8,13,24]

Figure S8 Adverse Events (RCTs)

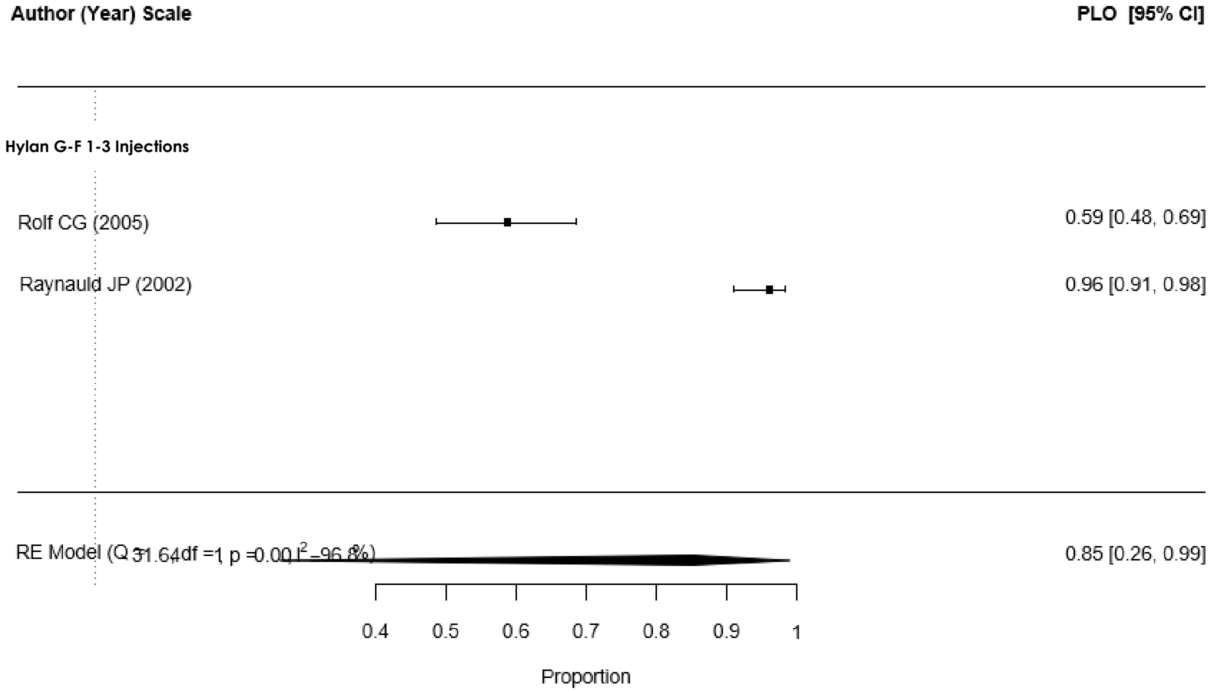

Across 2 RCTs with 217 participants, 85% (95% CI 26, 99; p=0.2174) of those that used Hylan G-F 1-3 weekly injections experienced an adverse event.[18,20]

Figure S9 Serious Adverse Events (Non-RCTs)

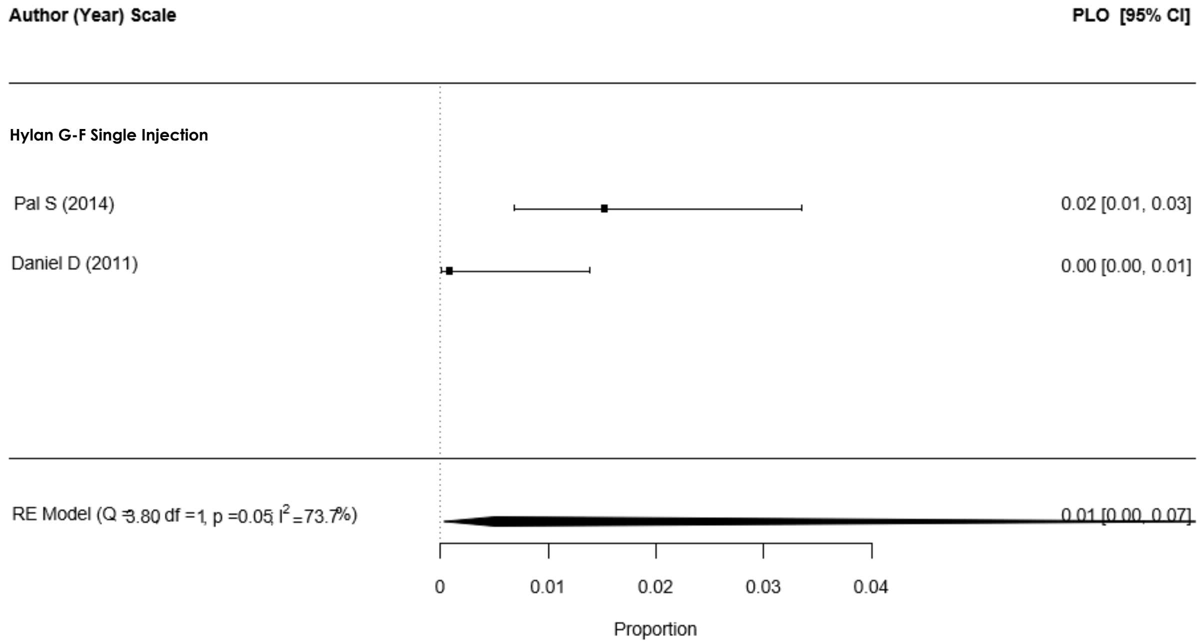

Across 2 non-RCT studies with 965 participants, 1% (95% CI 0, 7; p=0.0002) of those that used single Hylan G-F injection experienced a serious adverse event.[8,15]

Figure S10 Serious Adverse Events (RCTs)

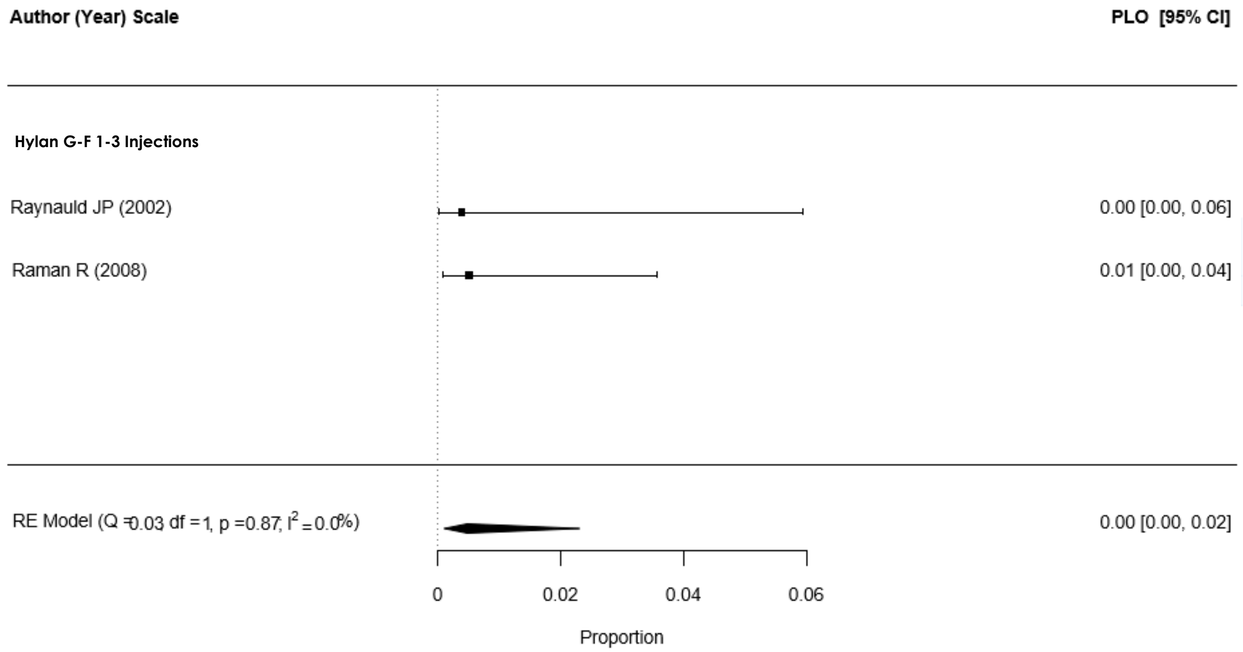

Across 2 RCTs with 321 participants, 0% (95% CI 0, 2; p<0.0001) of those that used Hylan G-F 1-3 weekly injections or experienced a serious adverse event.[17,18]

Figure S11 Treatment-Related Adverse Events (Non-RCTs)

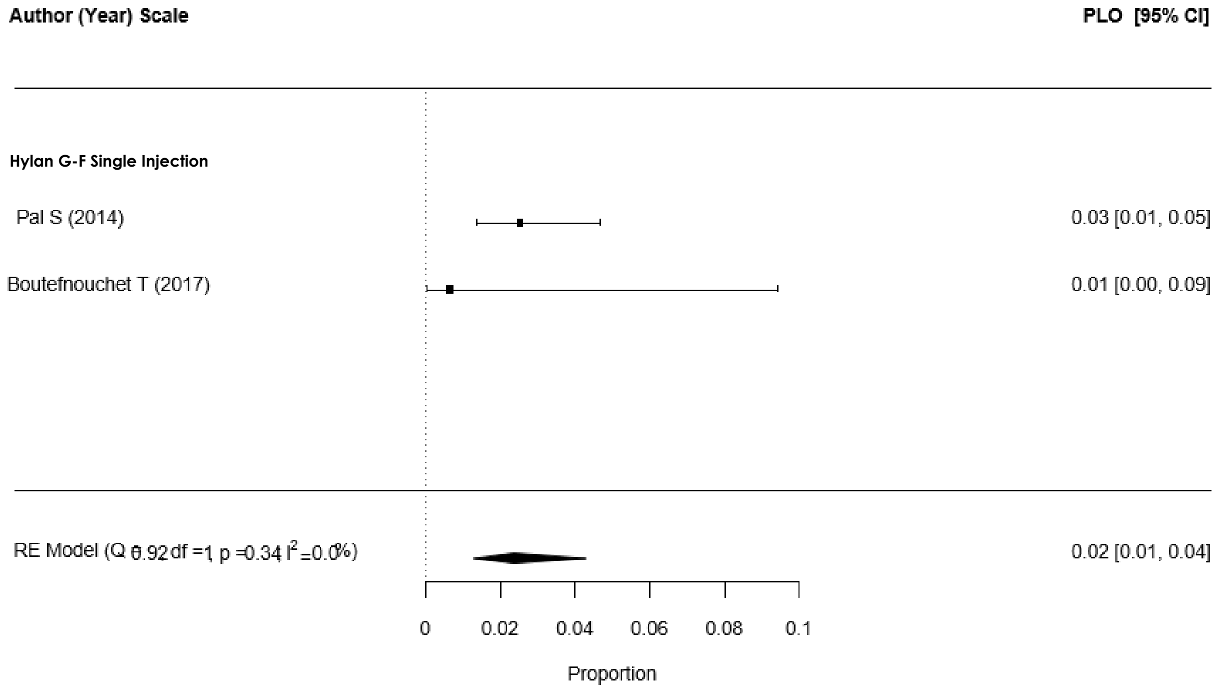

Across 2 non-RCT studies with 471 participants, 2% (95% CI 1, 4; p<0.0001) of those that used single Hylan G-F injection experienced a treatment-related adverse event.[5,15]

Figure S12 Treatment-Related Adverse Events (RCTs)

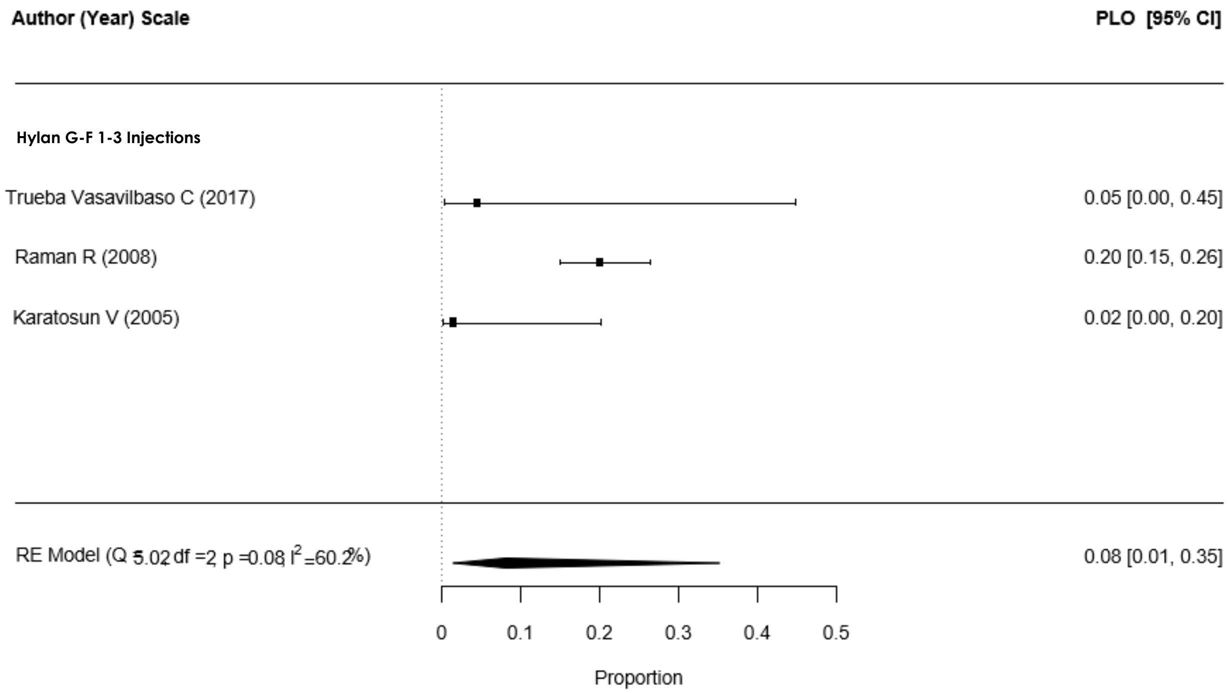

Across 3 RCTs with 236 participants, 8% (95% CI 1, 35; p=0.0088) of those that used Hylan G-F 1-3 weekly injections experienced a treatment-related adverse event.[11,17,21]
